# Synthesis and Hybrid SAR Property Modeling of Novel Cholinesterase Inhibitors †

**DOI:** 10.3390/ijms22073444

**Published:** 2021-03-26

**Authors:** Jiri Kos, Violetta Kozik, Dominika Pindjakova, Timotej Jankech, Adam Smolinski, Sarka Stepankova, Jan Hosek, Michal Oravec, Josef Jampilek, Andrzej Bak

**Affiliations:** 1 Regional Centre of Advanced Technologies and Materials, Czech Advanced Technology and Research Institute, Palacky University, Slechtitelu 27, 78371 Olomouc, Czech Republic; josef.jampilek@gmail.com; 2 Department of Chemistry, University of Silesia, Szkolna 9, 40007 Katowice, Poland; violetta.kozik@us.edu.pl; 3 Department of Analytical Chemistry, Faculty of Natural Sciences, Comenius University, Ilkovicova 6, 84215 Bratislava, Slovakia; pindjakova.dominika@gmail.com (D.P.); timotej.jankech@gmail.com (T.J.); 4 NT-LAB o.z., Teplicka 35, 92101 Piestany, Slovakia; 5 GiG Research Institute, Pl. Gwarkow 1, 40166 Katowice, Poland; smolin@gig.katowice.pl; 6 Department of Biological and Biochemical Sciences, Faculty of Chemical Technology, University of Pardubice, Studentska 573, 53210 Pardubice, Czech Republic; sarka.stepankova@upce.cz; 7 Department of Pharmacology and Toxicology, Veterinary Research Institute, Hudcova 296/70, 62100 Brno, Czech Republic; hosek.jan@vri.cz; 8 Global Change Research Institute CAS, Belidla 986/4a, 60300 Brno, Czech Republic; oravec.m@czechglobe.cz

**Keywords:** 4-aminosalicylanilides, carbamate synthesis, lipophilicity, cholinesterase inhibition, CoMSA, molecular docking, similarity-activity landscape index

## Abstract

A library of novel 4-{[(benzyloxy)carbonyl]amino}-2-hydroxybenzoic acid amides was designed and synthesized in order to provide potential acetyl- and butyrylcholinesterase (AChE/BChE) inhibitors; the in vitro inhibitory profile and selectivity index were specified. Benzyl(3-hydroxy-4-{[2-(trifluoromethoxy)phenyl]carbamoyl}phenyl)carbamate was the best AChE inhibitor with the inhibitory concentration of IC_50_ = 36.05 µM in the series, while benzyl{3-hydroxy-4-[(2-methoxyphenyl)carbamoyl]phenyl}-carbamate was the most potent BChE inhibitor (IC_50_ = 22.23 µM) with the highest selectivity for BChE (SI = 2.26). The cytotoxic effect was evaluated in vitro for promising AChE/BChE inhibitors. The newly synthesized adducts were subjected to the quantitative shape comparison with the generation of an averaged pharmacophore pattern. Noticeably, three pairs of fairly similar fluorine/bromine-containing compounds can potentially form the activity cliff that is manifested formally by high structure–activity landscape index (SALI) numerical values. The molecular docking study was conducted for the most potent AChE/BChE inhibitors, indicating that the hydrophobic interactions were overwhelmingly generated with Gln119, Asp70, Pro285, Thr120, and Trp82 aminoacid residues, while the hydrogen bond (HB)-donor ones were dominated with Thr120. π-stacking interactions were specified with the Trp82 aminoacid residue of chain A as well. Finally, the stability of chosen liganded enzymatic systems was assessed using the molecular dynamic simulations. An attempt was made to explain the noted differences of the selectivity index for the most potent molecules, especially those bearing unsubstituted and fluorinated methoxy group.

## 1. Introduction

As is known, cholinesterase (ChE) lyses choline-related esters that can serve as cholinergic neurotransmitters [1]. Roughly speaking, two groups of serine hydrolases are present in the human nervous system: acetyl-based (AChE) and butyryl-based (BChE) cholinesterases, respectively [2]. Unfortunately, the progressive degeneration of the central nervous system (CNS) with dysfunction of perception/cognition and dementia symptoms is characteristic for Alzheimer’s disease (AD) [3]. Following the cholinergic hypothesis, the potential cholinesterase inhibitors (ChEIs) are postulated to reduce the psychosomatic AD disorders [4]. In fact, the competitive and non-competitive ChEIs are used as marketed drugs in the AD pharmacotherapy (e.g., galanthamine, rivastigmine, tacrine, donepezil) in order to diminish the disease symptoms [5,6,7,8].

Diversely substituted amide (-CONH-) or carbamate (-OCONH-) fragments can be incorporated as privileged structural motifs (scaffolds) in the design of the potential anti-ChE agents [9,10,11,12]. As a matter of fact, the amide/carbamate-based groups are present as building blocks or linkers in vast number of clinically implemented drugs [13,14,15]. Thus, the amide/carbamate-like anti-AD derivatives appear to be an attractive synthetic and modeling target as well.

The pharmacological property profiling and potency modeling strategies are commonly utilized to escort the synthetic efforts and nominate the prospective candidates in the hit→lead→seed→drug race [16]. SAR-guided (structure–activity) exploration of the descriptor-based feature space seems pivotal at the crucial decision-making phases of drug discovery [17]. Hence, in silico computation of the ADMET-tailored properties (Absorption, Distribution, Metabolism, Excretion, Toxicity) is expected to minimize the probability of drugs’ late attrition [18]. As a matter of fact, a range of meaningful computer-supported approaches have been proposed to restrict the values of structural and/or physicochemical descriptors to the ADMET-friendly property space [19]. Basically, in silico-assisted approximation of the host–guest intermolecular recognition patterns can be dichotomized into the ligand-based and structure-related methodologies. In practice, the collaborative fusion of direct (receptor-dependent) and indirect (receptor-independent) procedures seems advisable in namely consensus approach for searching of promising drug molecule [20]. Conceptually, the mapping of the binding/active site in the pharmacophore-guided study is based on the simple tenet of substituent interchangeability and complementarity in the congeneric (structurally related) series of compounds. It is assumed that similar size, shape, and charge distributions should exert a comparable impact on the binding affinity and pharmacological profile, respectively [21]. Practically, the similarity-driven methodologies have been engaged in the comparable molecular shape analysis (e.g., CoMSA), where machine learning techniques are coupled with descriptor elimination/selection algorithms in order to approximate roughly the complex biological reality [22]. Despite some obstacles, the distance-related similarity investigations between structurally-alike molecules contribute significantly in the multidimensional quantitative (mD-Q) SAR practice [23]. Moreover, the numerical estimation of the ADMET-friendly features conjugated with desirable drug affinity (structure–activity landscape index, SALI) produces the SAR landscape, where the smooth/flat (homogenous) areas are striated with sharp (heterogeneous) activity cliffs [24]. Obviously, the clever management of SALI-related information can provide hints, which might be incorporated at the synthetic stage in order to modulate pharmacological effects and/or demolish unwanted side effects [25].

The relatedness of a ligand steric/electronic/lipophilic pattern can be also mediated by a complementary target surface using the protein-based docking procedures, where the (bio)effector-binding mode is determined according to the atomic coordinates of both host and guest molecules [26]. Despite drugs being basically engineered to fit tightly into the active interface of the enzyme/receptor, the binding goodness evaluation is continually questionable due to the lack of truly selective scoring functions [27]. Since the ligand–enzyme interaction pattern is dependent on their conformations and relative orientations (poses), it seems advisable to investigate the guest–host system stability using molecular dynamic simulations (MDs) [28].

The primary objective of the presented study was the design, synthesis, and in vitro specification of the anti-AChE/BChE profile for the new set of ring-substituted benzyl[4-(arylcarbamoyl)phenyl-3-hydroxy]carbamates carrying different functionalized aryl side chains. Moreover, the structured in silico computation of ADMET-friendly property was performed to estimate the intermolecular similarity, lipophilicity, and inhibitory potential, respectively. The newly synthesized adducts were subjected to the quantitative shape comparison with the generation of an averaged pharmacophore pattern. The molecular docking approach was employed for the most potent AChE/BChE inhibitors in order to procure an additional comprehension of the binding mode. Finally, the stability of some fused ligand–enzyme systems was evaluated using the molecular dynamic simulations. An attempt was made to explain the observed variations of the selectivity index for the potent molecules.

## 2. Results and Discussion

### 2.1. Design and Synthesis

**Scheme 1:**
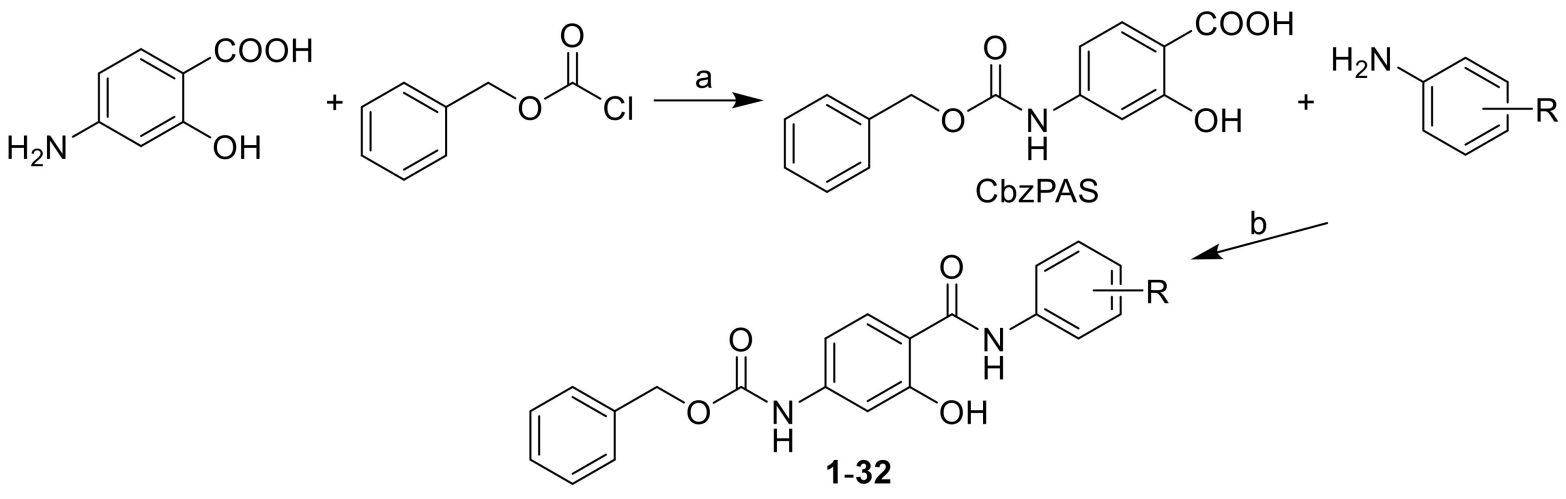
Synthesis of 4-{[(benzyloxy)carbonyl]amino}-2-hydroxybenzoic acid (CbzPAS) and corresponding anilides 1–32. *Reagents and conditions:* (**a**) MeOH, NaHCO_3_, room temperature, 24 h; (**b**) PCl_3_, chlorobenzene, microwave reactor, 130 °C, 30 min.

The synthesis of the carbamates of 4-aminosalicylanilides was carried out in two steps (see Scheme 1). The primary amino moiety of 4-aminosalicylic acid was protected by a reaction with benzyl chloroformate in an alkaline medium to form 4-{[(benzyloxy)carbonyl]amino}-2-hydroxybenzoic acid (CbzPAS) that was subsequently condensed with an appropriate substituted anilines using phosphorus trichloride in dry chlorobenzene under microwave conditions to give a series of investigated benzyl carbamates of 4-aminosalicylanilides.

**Figure 1:**
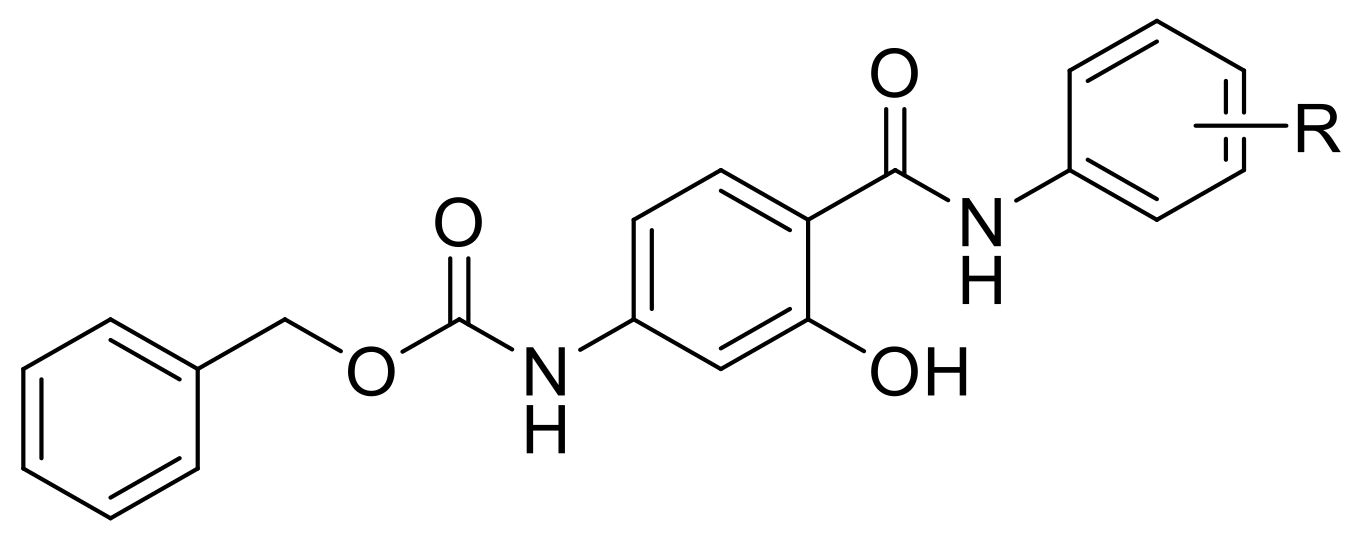
Structure of ring-substituted benzyl[4-(arylcarbamoyl)phenyl-3-hydroxy]carbamates **1**–**32**.

The lipophilicity of all the target compounds was investigated by means of RP-HPLC determination of capacity factors *k* with a subsequent calculation of log *k*. The retention times of individual compounds were determined under isocratic conditions with methanol as an organic modifier in the mobile phase using end-capped non-polar C18 stationary RP columns. All the results are shown in Figure 1 and Table 1.

### 2.2. Probability-Guided Pharmacophore Mapping

All synthesized ring-substituted benzyl[4-(arylcarbamoyl)phenyl-3-hydroxy]-carbamates **1**–**32** were tested to specify their in vitro inhibitory profile toward AChE and BChE targets and were compared with marketed drugs rivastigmine (RIV), galanthamine (GLT), and the pattern acid CbzPAS. Despite different mechanisms of action, RIV and GLT are the 2nd generation of ChEIs that pseudo-irreversibly inhibit AChE and BChE [8,14], respectively. Table 1 reports the IC_50_ values (μM) that express the quantitative measure of inhibitor concentration needed to reduce the biological process by 50%.

Benzyl(3-hydroxy-4-{[2-(trifluoromethoxy)phenyl]carbamoyl}phenyl)carbamate (**15**) showed the best AChE inhibition (IC_50_ = 36.05 µM), while benzyl{3-hydroxy-4-[(2-methoxyphenyl)carbamoyl]phenyl}carbamate (**2**) was the most potent BChE inhibitor (IC_50_ = 22.23 µM). In addition, compound **2** demonstrated the highest selectivity index for BChE (SI = 2.26). In addition, carbamates **12** (R = 2-CF_3_) and 28 (R = 2,4,6-F) showed BChE inhibition IC_50_ values of 33.59 and 31.03 µM, respectively and SI 1.68 and 1.61, respectively.

**Figure 2:**
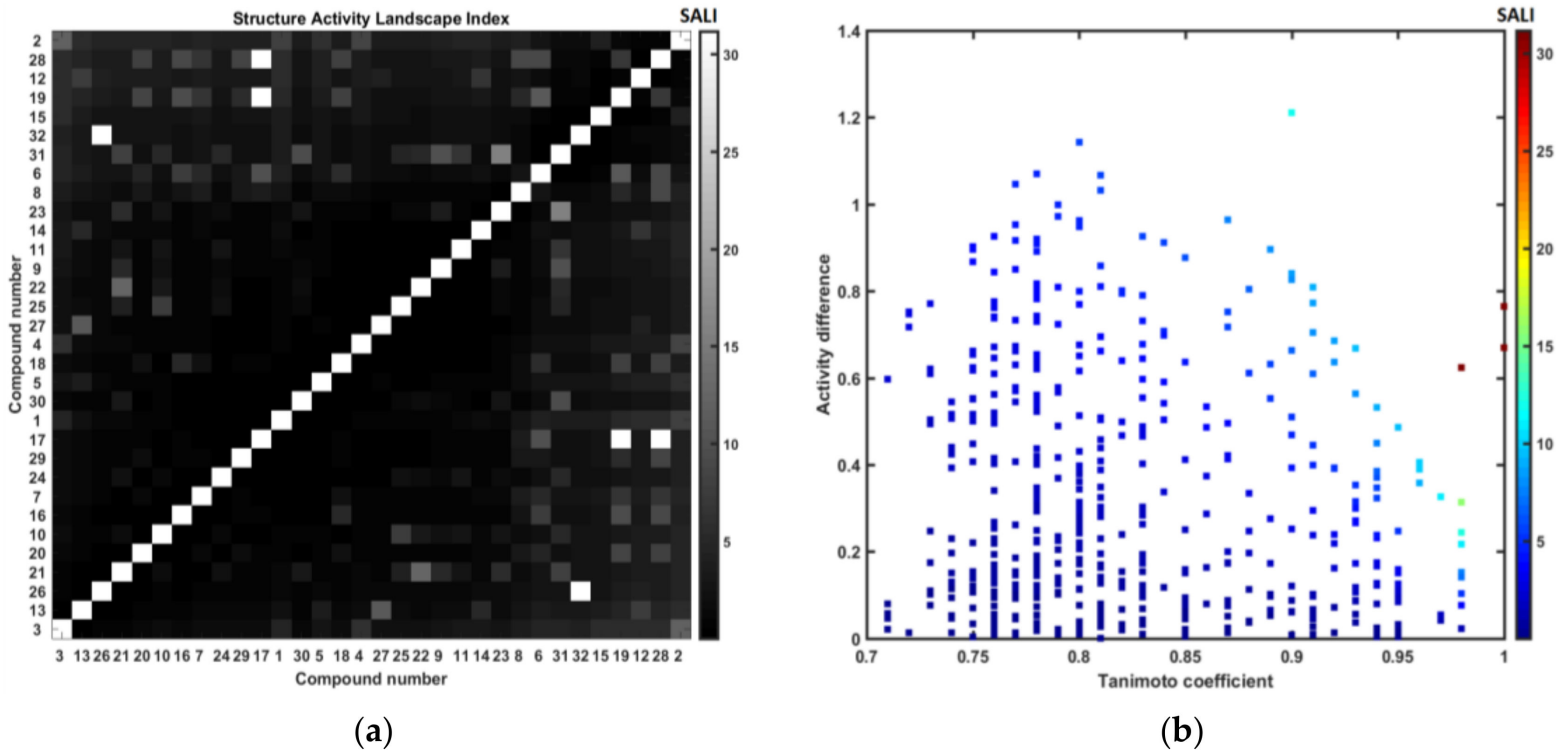
Gray-scaled structure–activity landscape index SALI plane with molecules 1–32 ordered according to increasing BChE inhibitory potential (**a**) and neighborhood plot (**b**).

A comprehensive visualization tool for the investigation of SAR trends was provided in the form of the structure–activity landscape indexes (SALI), where the planar image of molecular similarity is augmented with molecular activity profile [29]. Basically, the detection of the continuity areas and/or activity cliffs depends largely on the accessibility of structurally related compounds (chemotypes) with noticeable variations in the activity profile. The distribution of the Tanimoto coefficients was analyzed for the triangular T_32×32_ matrix revealing that the majority of compounds are characterized by pretty high similarity metric (0.85 ≥ T ≥ 0.75). Interestingly, the potent compounds 2 and 15 are marked as fairly similar with T = 0.93. Obviously, for structurally related molecules (e.g., stereoisomers T→1), SALI→ infinity; therefore it is recommended to replace such values by the largest SALI values [30]. The symmetrical grayscaled heatmap of SALI values for the congeneric series of analyzed compounds is illustrated in Figure 2a, where axes correspond to molecules sorted according to the increasing pBChE inhibitory potential (ΔpBChE = 1.2), respectively. Accordingly, the white spot of the heatmap represent the highest numerical values of SALI parameters, while the black ones specify the minimal ones. In fact, molecule **15** is characterized by low values of SALI indexes with the remaining compounds in the dataset, while the most potent compound **2** is accompanied by gray pots of the inactive molecules **3**, **4** (positional isomers) and **1** (unsubstituted analogue), respectively. The structural modifications that considerably affect the molecular activity profile are illustrated as the neighborhood plot in Figure 2b, where the structural relatedness between pairs of compounds is shown in the function of the activity difference and color-coded according to SALI values. Noticeably, three pairs of fairy similar fluorine/bromine-containing compounds (**17** vs. **19** and **28**, **26** vs. **32**) can potentially form the activity cliff that is manifested formally by high SALI numerical values (see Figure 2b). In consequence, further dense samplings of the indicated SAR-variations (T > 0.95 & ΔpBChE > 1) seem reasonable for the investigated set of BChE inhibitors.

**Figure 3:**
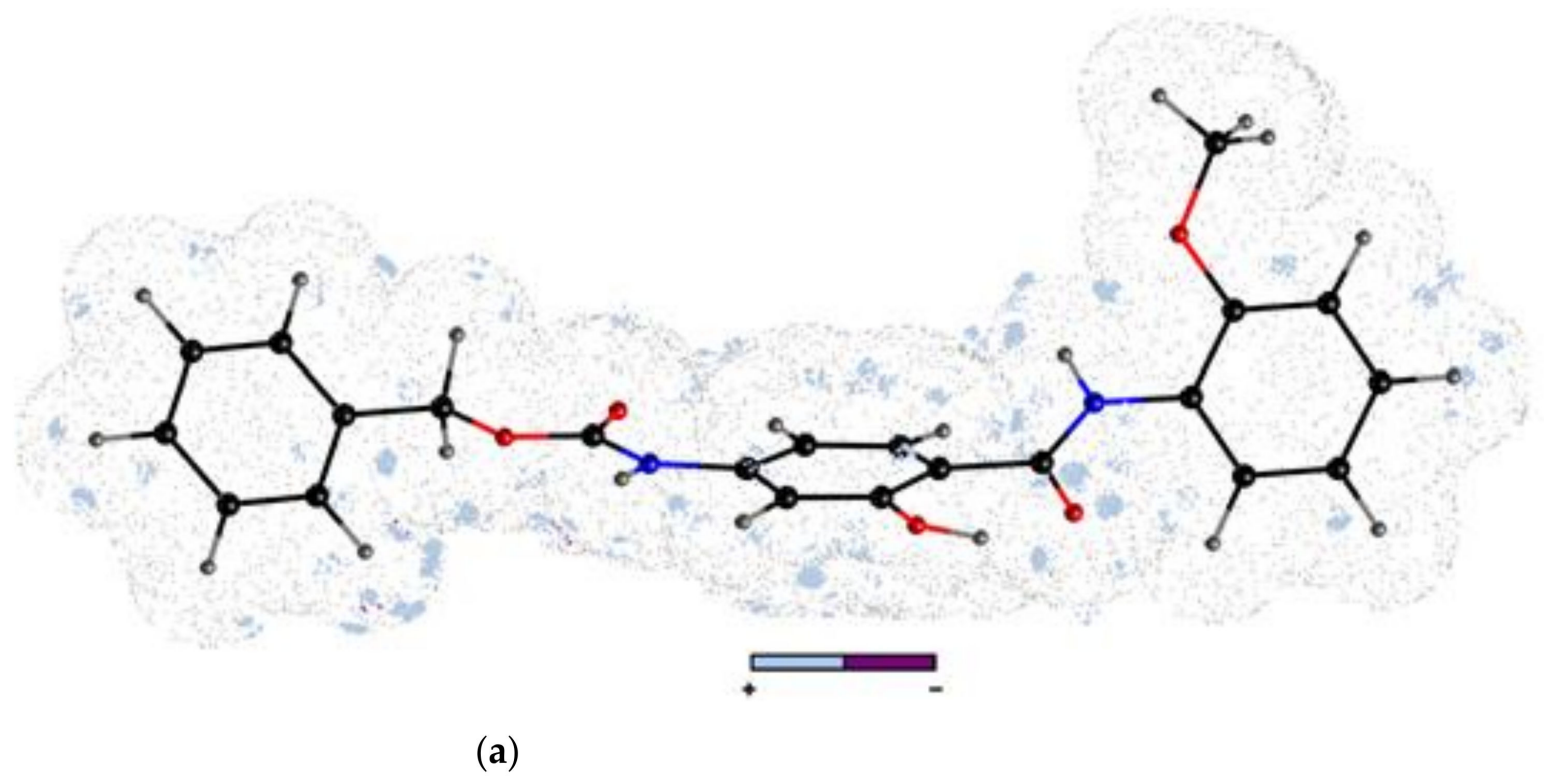

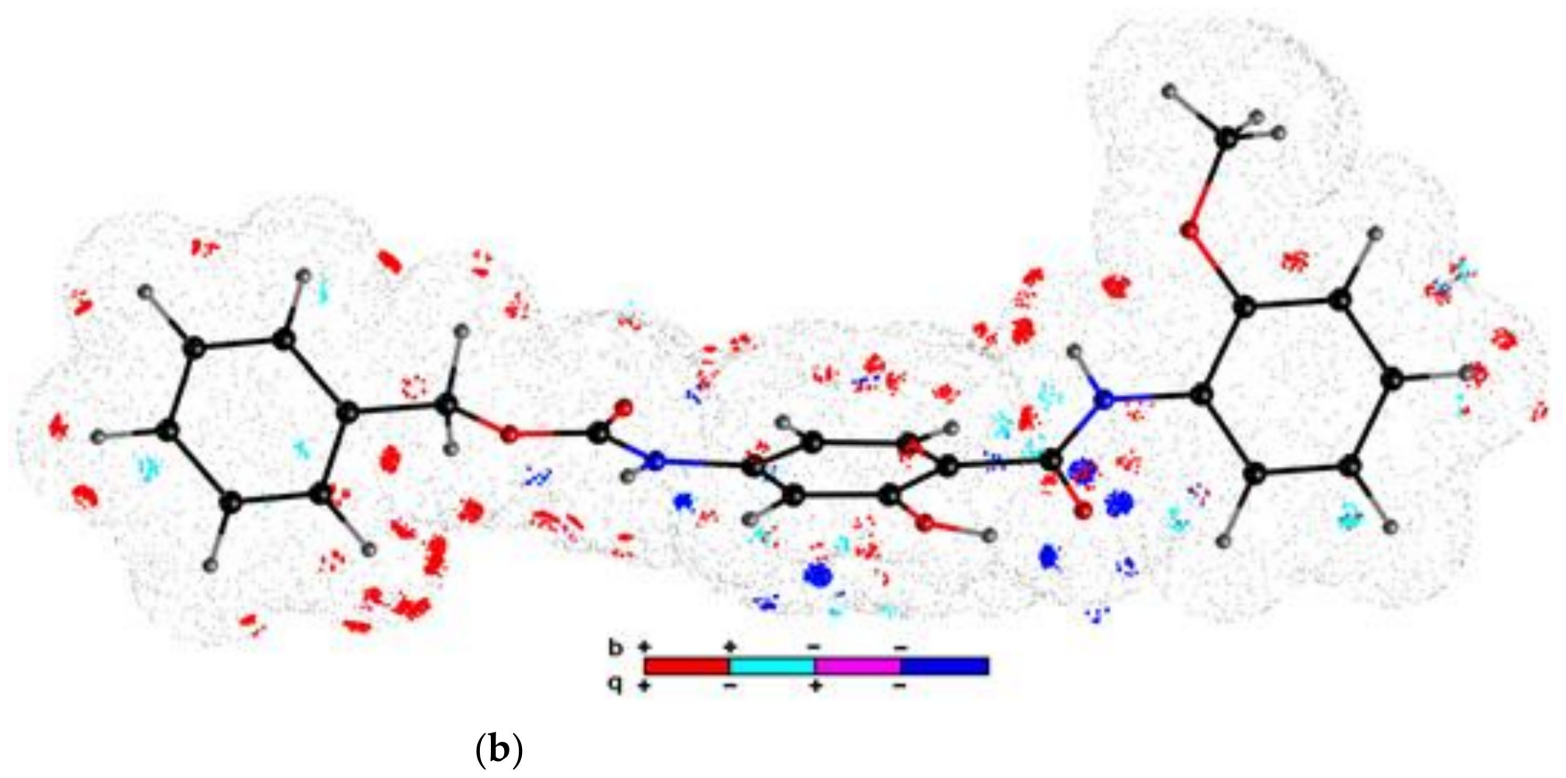
Three-dimensional (3D) pattern specified by comparable molecular shape analysis (CoMSA) IVE (iterative variable elimination-partial least square)-PLS procedure for 24/8 training/test samplings of potential BChE inhibitors. Colors code the contribution (**a**) and four possible combinations of mean charge descriptor and correlation coefficient (**b**). The potent compound 2 is plotted as a reference molecule.

The crucial distributions of the electronic/steric/lipophilic properties of the guest–host composition can be specified by the systematic sampling of the functional group relatedness with the introduction of the pharmacophore concept in QSAR studies [31]. In reality, the spatial arrangement of pharmacophoric features is determined for the homogeneous ensemble of molecules that share the common structural pattern (chemotype), because the enhancement of the SAR applicability domain (AD) for a non-congeneric set of structures is a rather elusive and enigmatic operation. Moreover, the prediction of the modeled property is firmly dependent on the molecule training and test separation. On the other hand, there are no strictly defined rules for splitting the set of compounds into training/test subfamilies in order to maintain the model robustness as well as its predictive power. Hence, the recurrent and interchangeable training/test subset division was proposed for the probability-driven pharmacophore mapping based on the stochastic model validation (SMV) algorithm [32]. Despite the CPU-intense (Central Processing Unit) SAR calculations, the whole pool of systematically produced training/test subpopulations (ratio 3:1) was investigated in CoMSA modeling of the BChE inhibition potency ($C_{32}^{8}$ ≈ 10^7^ models). The frequency distribution in the test set for preferable statistic parameters of model robustness ($q_{cv}^{2}\;$ > 0.6 and $q_{test}^{2}\;$ > 0.5) indicted that the potent compound 2 is noticeably overrepresented in contrast to molecule **15**. Surprisingly, the preferential selection of the active compounds (**2** and **6**) and inactive ones (**3**, **13**, **23** and **26**) that are basically *ortho*- and *meta*-substituted analogues resulted in the generation of the robust models with the acceptable predictive power for the test set. Finally, the selection-oriented pharmacophore pattern was generated based on the robust models following the principles of IVE (iterative variable elimination-partial least squares)-PLS algorithm that is described elsewhere [33]. The graphical representation of the descriptor-based areas that contribute (un)favorably into CoMSA models is shown in Figure 3. The relative contribution of the surface/charge descriptors is weighted by the corresponding regression coefficient indicating the regions of the positive (bright color) and negative (dark color) impact on the inhibitory potency (see Figure 3a). Moreover, the four possible combinations of the charge and the mean regression coefficients are introduced in Figure 3b.

Noticeably, a diverse set of steric and electrostatic features was indicated on the averaged surface for the enzyme-selective ligands; therefore, the direct translation of the pharmacophore-based areas of the space into the complementary pseudoreceptor model that potentially harbors putative inhibitors is a fairy complicated task. The spatial pattern plotted in Figure 3a indicates the favorable steric contributions of areas that spread uniformly over the 4-{[(benzyloxy)carbonyl]amino}-2-hydroxybenzoic acid skeleton and aniline-like substituent as well. Not surprisingly, the positively charged areas of hydrogen atoms directly attached to nitrogen atoms and the negatively charged oxygen atoms of carbonyl groups were depicted as the favorable contributors to the inhibitory potency of the investigated compounds (see Figure 3b). The specified atoms can potentially form the hydrogen bonds with the hypothetical receptor aminoacid residue in the enzyme active site. Unfortunately, the provided pharmacophoric pattern does not explain the observed variations in selectivity of molecule 2 and 15 toward A/BChE enzymes. In fact, no pharmacophoric areas were specifically pointed out as dominant ones to increase the inhibitory potency; however, the consensus 3D-QSAR modeling provides the spatial map of steric/electronic features of the ligand-site composition.

### 2.3. Similarity-Driven Property Assesment

**Figure 4:**
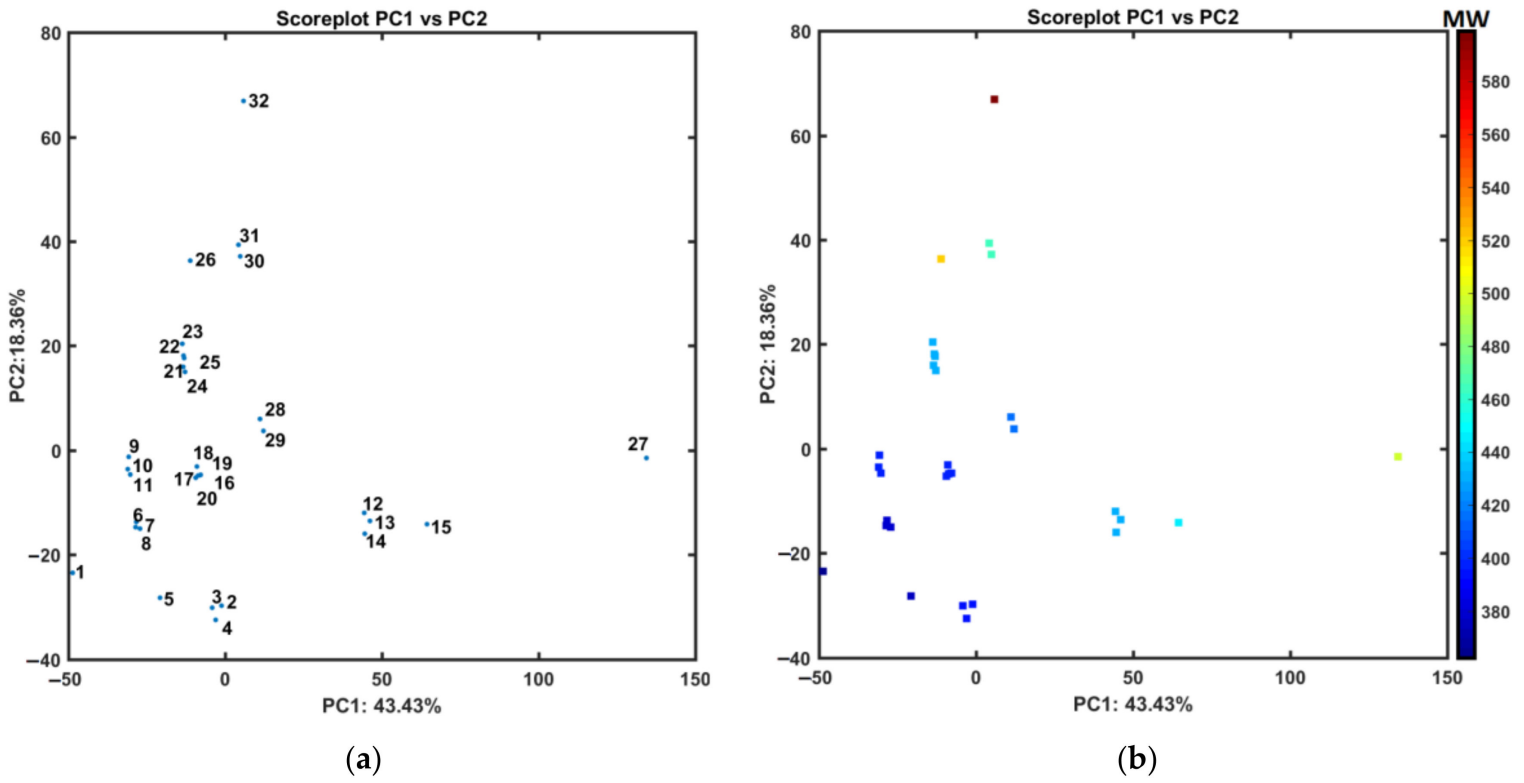
Projection of compounds 1–32 (**a**) with corresponding molecular weights (**b**) on plane defined by PC1 and PC2. Colors code molecular weight (MW) values.

The distance-oriented property evaluation was conducted using the Principal Component Analysis (PCA) and Hierarchical Clustering Analysis (HCA) on the pool of 2819 descriptors derived from Dragon 6.0 software. The descriptor-based data were organized into a matrix X_32×2819_ with rows presenting molecules (objects) and columns representing numerical variables (parameters), respectively. The resulting dataset was centered and standardized, since the calculated parameters varied considerably. The number of significant principal components (PCs) was determined according to the percentage of the modeled variance. The first four PCs accounted for 77.6% of the total data variance, while the first three PCs described 72.42%. The respective scoreplots with the projection of molecules **1**–**32** and color-coded with the corresponding molecular weight (MW) on plane PC1 vs. PC2 are presented in Figure 4a,b.

The dissimilarities of molecules **12**–**14**, 27 with trifluoromethyl substituent(s) (-CF_3_) and compound 15 with trifluromethoxy group (-OCF_3_) from the remaining ones, are observed along the first principal component (PC1 > 45). Moreover, objects **2**–**4** that are methoxy-based (-OCH_3_) positional isomers and molecule **5** with methyl substituent (-CH_3_) are also clustered together along the second principal component (PC2 <−20). Interestingly, the lower weighted molecules (MW < 400) are located in the negative area of the plane (PC1 < 0 and PC2 < 0).

**Figure 5:**
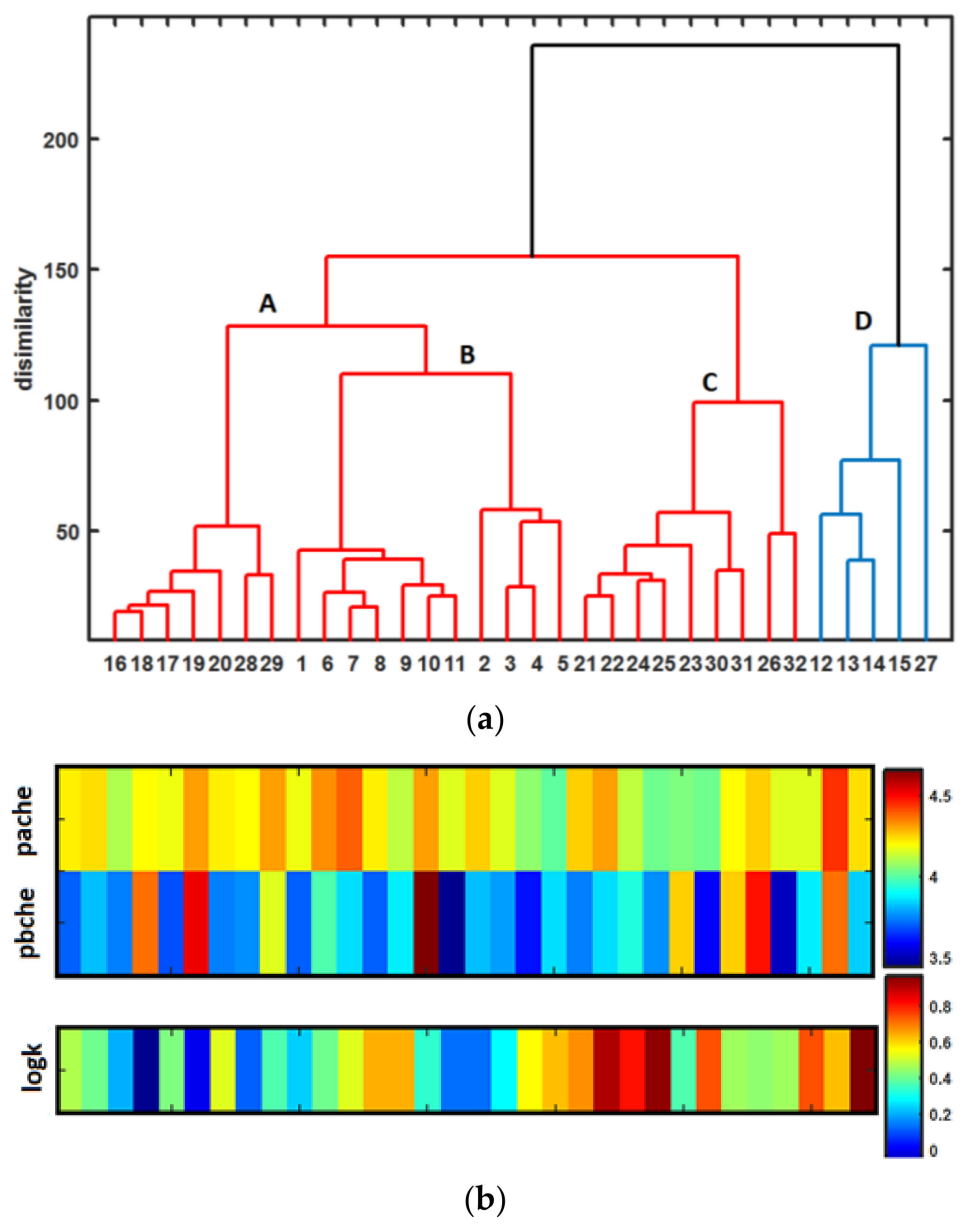
Dendogram of compounds **1**–**32** in descriptor-based space (**a**) and color map of experimental activity and lipophilicity values in logarithmic scale (**b**).

Moreover, the exploratory analysis of the descriptor-driven data structure was performed by tracing the (dis)similarities between objects (molecules) in the multidimensional variable space in order to reveal its clustering tendency. Due to its hierachical nature, the HCA method basically leads to sub-optimal clustering of objects that is mainly dependent on the procedure employed for clusters’ linkage [34]. The interpretability of the extracted data structure is not obvious in the high-dimensional variable space; therefore, the findings are presented in the form of dendogram constructed on the Euclidean-based distance and the Ward linkage algorithm, where the OX axis presents the sequence of objects/parameters and the OY axis specifies the dissimilarity [35]. The dendogram shown in Figure 5a proved our previous PCA findings (see Figure 4a), where at least three sub-clusters (A,B,C) can be distinguished—molecules **12**–**15** and **27** (blue lines) differ noticeably from the rest of the objects (red lines), respectively. The HCA dendogram was augmented with a color-coded map of the experimental activity and lipophilicity data in the logarithmic scale as presented in Figure 5b. As a matter of fact, a simultaneous interpretation of the dendogram objects (sorted according to the Ward linkage method) and the colored map of the empirical data revealed that there is no clear relationship between the investigated data. Roughly speaking, the molecules grouped in cluster D are characterized by relatively high values of the log *k* parameter when compared to the remaining objects.

**Figure 6:**
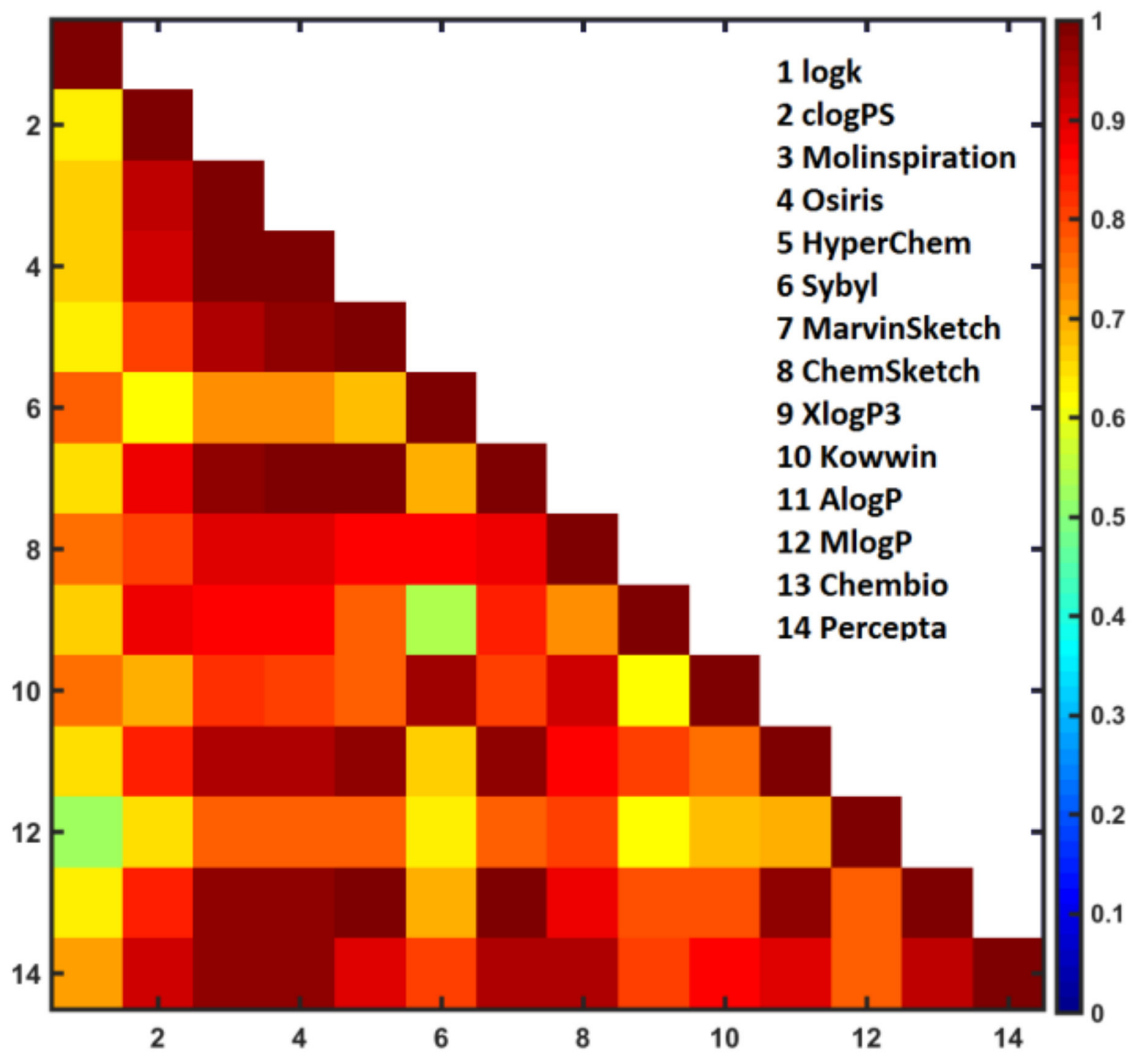
Matrix of linear correlation coefficients between empirical log *k* and clogP calculated by various programs.

From the user’s perspective, the non-trivial question arises of how to single out the best-suited method or combination of methods for clogP estimation of new compounds. In order to investigate the lipophilic profile of the analyzed molecules, the experimentally specified log *k* parameters were compared with the calculated lipophilicity coefficients (clogP) estimated using a set of in silico predictors, e.g., clogP, Molinspiration, Osiris, HyperChem 7.0, Sybyl-X, MarvinSketch 15, ACD/ChemSketch 2015, Dragon 6.0, Kowwin, XlogP3, ChemDraw, and ACD/Percepta programs (see Table S1 in the Supplementary Materials). Moreover, the selected physicochemical properties of rivastigmine, galanthamine, and compound 2 were predicted by ACD/Percepta 2012 (see Table S2 in Supplementary Materials). The corresponding clogP estimators deduced by the group of alternative programs were cross-compared with the experimental potency values and inter-correlated with each other, as illustrated in Figure 6. Relatively good correlations (*r* = 0.77) between the generated clogP values using Sybyl-X program and the empirical log *k* were recorded. The averaged clogP values over the set of programs produced the value of *r* = 0.73, whereas the median resulted in *r* = 0.70 with the experimental data.

On the whole, a pretty high inter-correlation was observed among the programs for lipophlicity specification (see Figure 6); however, some variations in clogP values resulted probably from different computational algorithms (atom/fragment- or descriptor-based) implemented in the software and/or training data engaged at the training stage—models are as good as the modeling data used. Additionally, the iterative variable elimination procedure (IVE-PLS) was applied on the integrated clogP matrix (X_32 × 13_) and log *k* parameter indicating Sybyl-X and XlogP3 estimators as significant contributors to the linear quantitative structure-property relationship QSPR model in namely consensus lipophilicity determination [36]. Consequently, the averaged values of the indicated clogP estimators were correlated with log *k* values resulting in *r* = 0.80, because not only the best inter-correlated logP estimators were chosen. Similarly, the same programs were specified with the stepwise procedure implemented in Matlab environment.

### 2.4. Docking and Molecular Dynamics Simulations

The pharmacophore-driven (ligand-based) SAR approaches can be coupled with the site-directed (structure-based) docking procedures that position a potential bio-effector molecule within a protein binding/active. The complementary host–guest binding mode is deduced based on the target spatial arrangement of atoms with the engagement of feature/descriptor-matching algorithms, where the ligand property space is correspondingly mapped to the receptor steric, electrostatic, and/or lipophilic patterns. Unfortunately, it is still not clear how to directly relate the pharmacological or ADMET effects to enthalpically and/or enthropically favorable ligand–receptor modes and scoring descriptors. On the other hand, in silico attempts to reconstruct the drug–protein interactions using the molecular docking are commonly accepted as a complimentary approach to the traditional mD-QSAR ligand-based methods [37].

**Figure 7:**
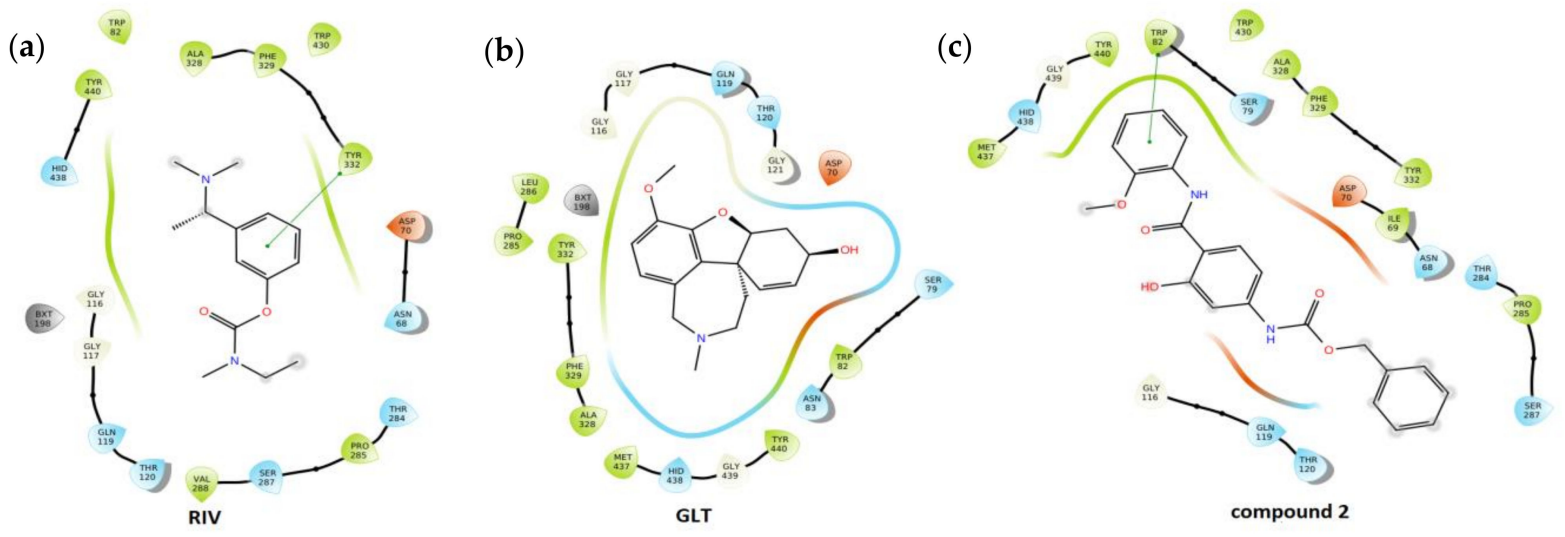
Planar comparison of binding RIV (**a**), GLT (**b**), and the most potent BChE inhibitor **2** (**c**) generated by the Maestro program.

**Figure 8:**
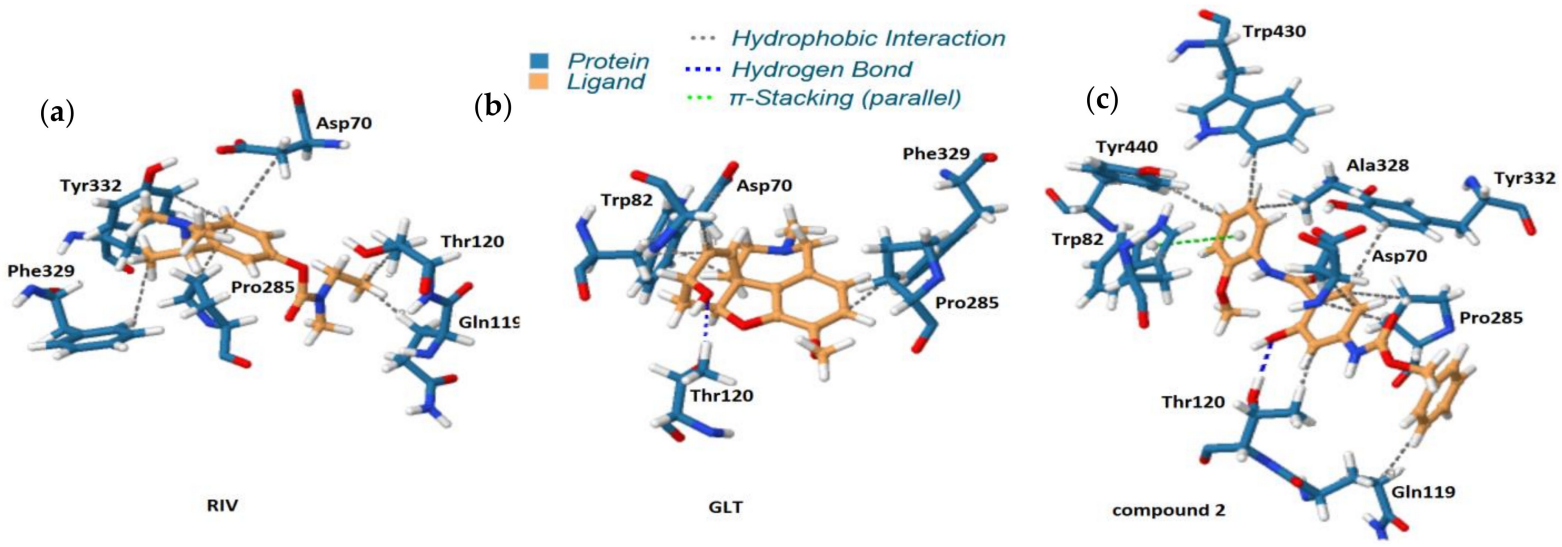
Spatial comparison of binding RIV (**a**), GLT (**b**), and the most potent BChE inhibitor **2** (**c**) generated by the PLIP program.

The structural geometries of human acetyl-/butyrylcholinesterase enzymes (A/BChE) in a complex with the commercially accessible drug molecules (e.g., galanthamine and rivastigmine) are being currently intensively scrutinized. The crystallographic structure of BChE determined at a sophisticated resolution of 2.6 Å in the ligand-containing state (holo) with the rivastigmine (RIV) analogue [3-[(1~{*R*})-1-(dimethylamino)ethyl]-4-oxidanyl-phenyl]~{*N*}-ethyl-~{*N*}-methyl-carbamate was retrieved from the Protein Data Bank repository (PDB code: 6eyf). Subsequently, the marketed drugs and the potential anti-BChE population (IC_50_ < 150) were docked in the active site of the enzymatic chain A using AutoDock Vina software in order to collate the binding mode of the analyzed analogues with the drug–enzyme interacting pattern [38]. The resulting molecular conformations and orientations (poses) of the potentially active compounds revealed mainly three types of non-binding interactions: the hydrogen bond (HB), hydrophobic, and π-stacking interactions, respectively. The graphical illustrations of the relevant ligand–enzyme interactions were produced for the entire population of the active compounds (IC_50_ < 150) using Schrödinger Maestro software and Protein–Ligand Interaction Profiler (PLIP) and compared with the commercial drug molecules (GLT and RIV) [39]. As a matter of fact, the hydrophobic interactions were overwhelmingly generated (70%) with Gln119 (20%), Asp70 (13.7%), Pro285 (13.7%), Thr120 (12.7%), and Trp82 (9.5%) aminoacid residues, while the HB-donor ones were dominated (50%) with Thr120, which confirms our previous findings [1,2]. π-stacking interactions were specified with Trp82 aminoacid residue of chain A as well. The planar (2D) and spatial (3D) binding pattern distribution for marketed drugs and the most active anti-BChE molecule **2** are presented in Figure 7 and Figure 8, respectively.

**Figure 9:**
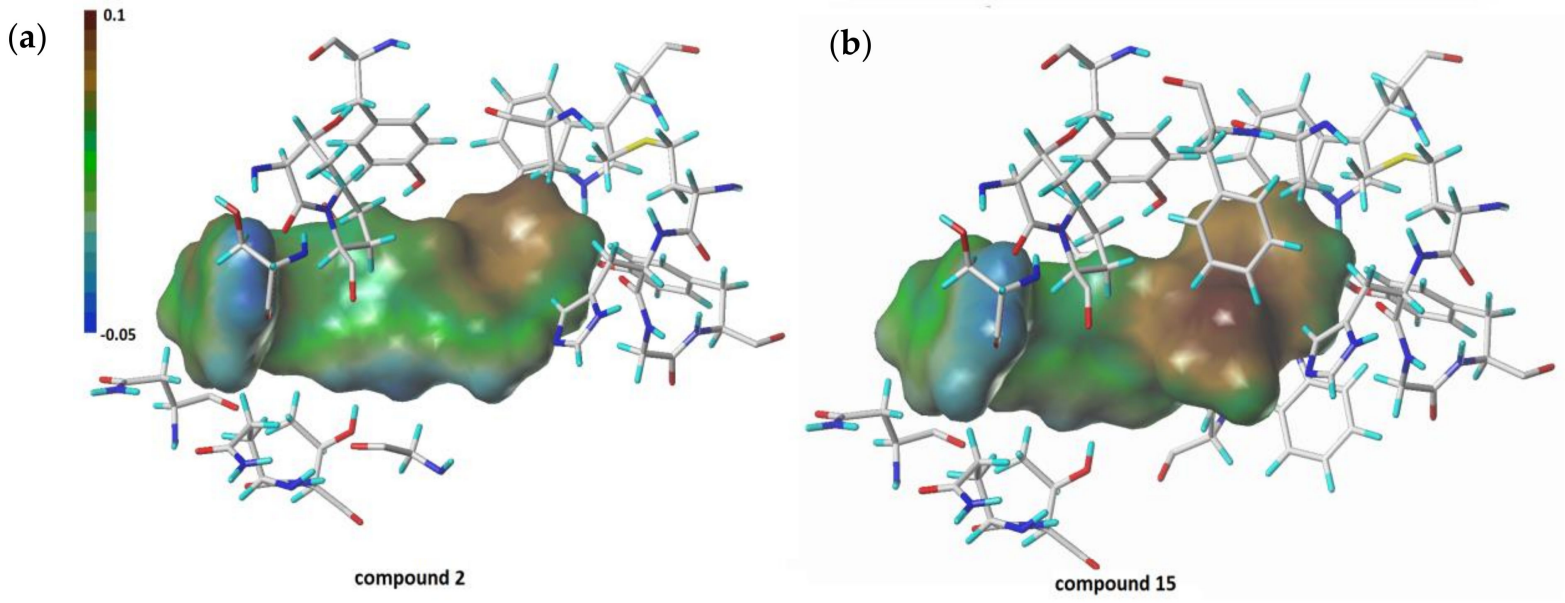
Lipophilic potential distribution on Connolly surface of molecule **2** (**a**) and **15** (**b**) with the residues of BChE active site.

The hydroxyl group of Thr120 seems to be the key structural motif in the formation of hydrogen bond (HBD) with hydroxyl oxygen of molecule **2** (see Figure 8c). The π-stacking interaction was revealed between the substituted anilide-based ring and Trp82, respectively. Unfortunately, the replacement of hydrogen atoms in the methoxy group of molecule **2** with fluorine in the trifluoromethoxy substituent in compound 15 does not change noticeably the enzyme–ligand binding pattern; however the additional hydrogen bond (HBA) is generated using the oxygen (acceptor) of Asn68 carbonyl group and nitrogen (donor) of molecule **15**. The molecular electrostatic potentials (MESP) as well as the lipophilic potentials determined on Connolly surface of molecule **2** and **15** were determined. In fact, the interrelation was reported previously between electrostatic and lipophilic potentials, because the molecular charge distribution affects the lipophilic property [40]. Noticeably, the electron-rich substituents correspond well to higher lipophilic potential values, as illustrated in Figure 9. Not surprisingly, the variations of charge distribution in molecule **2** and **15** have a visible impact on the lipophilic patterns, especially in the regions of –OCH_3_ and –OCF_3_ groups as indicated by brow colors (see Figure 9).

**Figure 10:**
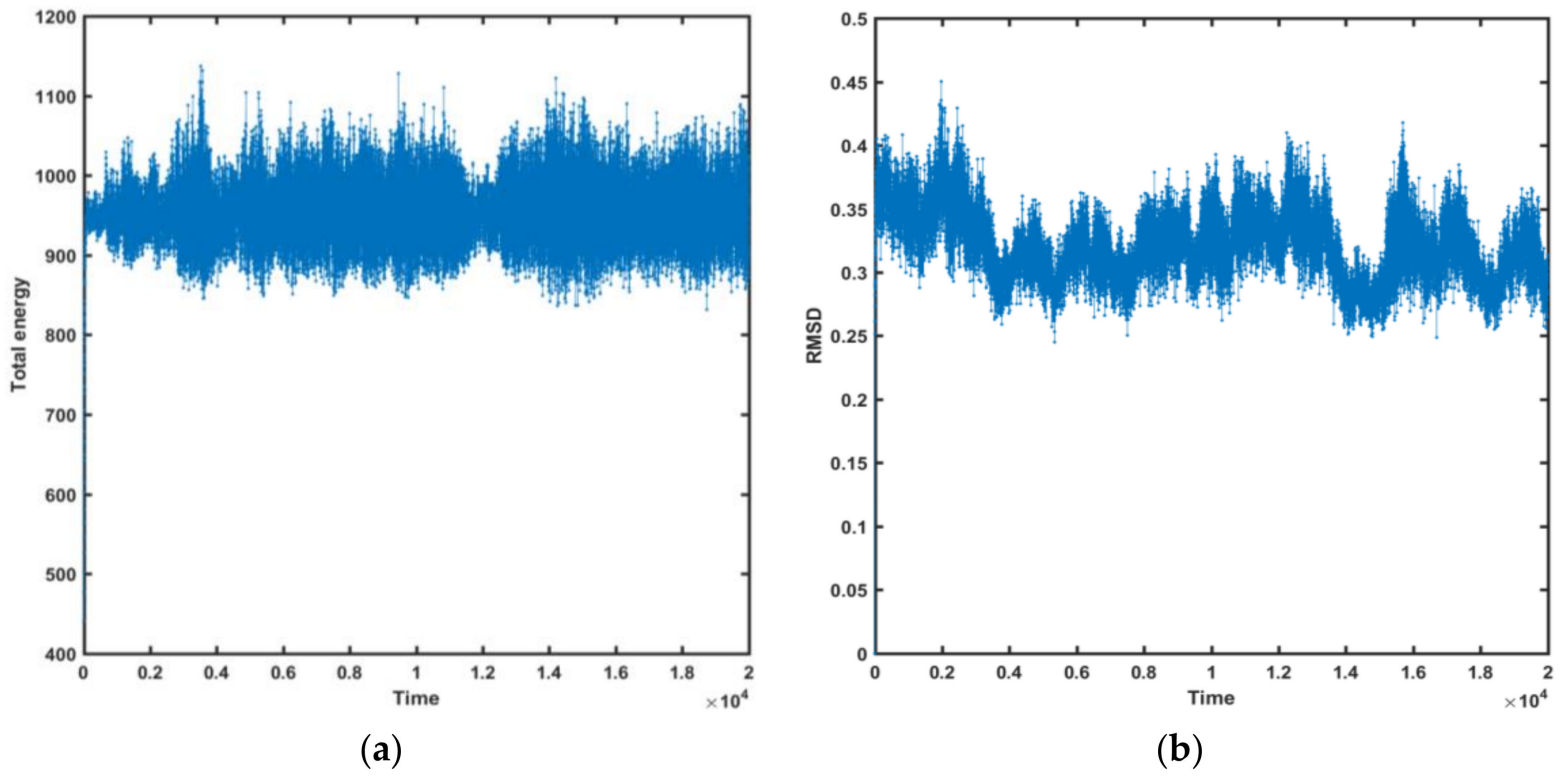
Total energy (**a**) and root mean square deviation (RMSD) (**b**) variations enzyme ‘bead’ atoms in RedMD simulations.

The molecular dynamic simulations (MDs) can supply fruitful knowledge regarding the host–guest complex stability as well as the potential mechanism of ligand action in the active site of the enzyme [41]. The liganded state (holo) of the BChE enzyme docked with the most potent molecule **2** was applied as the input structure in the MD simulations. Taking into account the technical feasibility (computational time and resource constraints), the CPU-intense MD calculations were conducted with the involvement of the flexible ligand and the rigid target according to the procedure implemented in Sybyl-X 2.2.1 program. Contrarily, the internal macromolecular motions (atom trajectories) and the system energy variations were investigated based on the reduced molecular dynamics (RedMD) algorithm. Accordingly, in the RedMD strategy, each aminoacid/nucleotide is expressed by a singular spherical particle (‘bead’) centered on a Cα or P atom with the mass that corresponds to the total mass of a given aminoacid or nucleotide [42,43,44]. The Berendsen algorithm was engaged in RedMDs on the NVT ensemble (constant number, volume, temperature) in order to control temperature. The initial atom velocities were assigned at 293K; however, the system was then heated, and the corresponding trajectories were specified at the temperature of 300K. The total length of the RedMDs was arranged to 20 ns with a 10 fs time step, respectively. The enzyme fluctuations of the total energy during the RedMD simulations are illustrated in Figure 10a. In fact, relatively small variations of the root mean square deviation (RMSD) values indicated the enzyme stability, as shown in Figure 10b.

**Figure 11:**
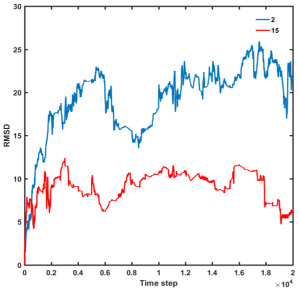
Variations of RMSD parameter of molecules **2** and **15**.

Noticeably, the side chains of compound **2** and **15** have an important impact on the resulting variations of RMSD values recorded for heavy atoms during MDs, as depicted in Figure 11. It seems that trifluoromethoxy substituent of molecule **15** is characterized by lower spatial flexibility compared to the methoxy group of compound **2**. Obviously, –OCF_3_ is considerably heavier and can generate greater steric hindrance in comparison to –OCH_3_. The most electronegative element fluorine can interact with the surrounding aminoacid residues that stiffen the ligand-enzyme system, as mirrored in Figure 11.

### 2.5. In Vitro Cell Viability Assay

To evaluate the potential cytotoxic effect, the preliminary in vitro screening of the antiproliferative activity of the most effective AChE/BChE inhibiting compounds **2** and **28** was performed. The human monocytic leukemia THP-1 cell line was selected for this purpose, as we described previously [45]. Nor **2** neither **28** show negative effect on relative cell viability up to the concentration of 30 µM. The relative cell viability was around 100% at this concentration. It makes compounds **2** and **28** interesting for further pharmacological evaluation and allows them to become lead compounds.

## 3. Materials and Methods

### 3.1. General Methods

All reagents were purchased from Merck (Sigma-Aldrich, St. Louis, MO, USA) and Alfa (Alfa-Aesar, Ward Hill, MA, USA). Reactions were performed using a Anton-Paar Monowave 50 microwave reactor (Graz, Austria). The melting points were determined on a Kofler hot-plate apparatus HMK (Franz Kustner Nacht KG, Dresden, Germany) and are uncorrected. Infrared (IR) spectra were recorded on Nicolet iS5 IR spectrometer (Thermo Scientific, West Palm Beach, FL, USA). The spectra were obtained by the accumulation of 256 scans with 2 cm^−1^ resolution in the region of 4000–450 cm^−1^. All ^1^H- and ^13^C-NMR spectra were recorded on a JEOL JNM-ECA 600II device (600 MHz for ^1^H and 150 MHz for ^13^C, JEOL, Tokyo, Japan) in dimethyl sulfoxide-d_6_ (DMSO-*d*_6_). ^1^H and ^13^C chemical shifts (δ) are reported in ppm. The purity of the final compounds was checked by the HPLC separation module Agilent 1200 series equipped with a DAD SL (Agilent Technologies, Santa Clara, CA, USA). The detection wavelength 210 nm was chosen with operational conditions described in Section 3.3. The solvent peaks in the (blank) chromatogram were deducted from the peaks in the chromatogram of the sample solution. The purity of individual compounds was determined based on the area of peaks in the chromatogram of the sample solution. High-resolution mass spectra were measured using a high-performance liquid chromatograph Dionex UltiMate^®^ 3000 (Thermo Scientific, West Palm Beach, FL, USA) coupled with an LTQ Orbitrap XL TM Hybrid Ion Trap-Orbitrap Fourier Transform Mass Spectrometer (Thermo Scientific) equipped with a HESI II (heated electrospray ionization) source in the positive mode.

### 3.2. Synthesis

4-{[(Benzyloxy)carbonyl]amino}-2-hydroxybenzoic acid (CbzPAS) [46]: 4-Aminosylicylic acid (6.0 g, 39.2 mM) was disolved in methanole (64 mL) at ambient temperature, and benzyl chloroformate (6.8 mL, 47.6 mM) was added dropwise. The reaction mixture was stirred at room temperature for the time period of 24 h. Then, the solvent was removed to dryness under reduced pressure. Crude product was dissolved in the mixture of ethyl acetate (200 mL) and 1M hydrochloric acid (200 mL). Layers were divided. The water layer was additionally extracted with ethyl acetate (3 × 50 mL). Afterwards, organic layers were collected and dried with magnesium sulfate. Then, the solvent was removed to dryness under reduced pressure. Yield 71%; Mp 219–220 °C; IR (ATR, cm^−1^): 1727, 1623, 1589, 1538, 1440, 1383, 1307, 1270, 1249, 1220, 1204, 1185, 1165, 1104, 1056, 1028, 998, 976, 905, 871, 774, 767, 760, 725, 691, 669, 656, 639, 620, 595, 573, 547, 509, 466, 453; ^1^H-NMR (DMSO-*d*_6_), δ: 11.39 (brs, 1*H*), 10.13 (s, 1*H*), 7.69 (d, *J* = 8.7 Hz, 1*H*), 7.46–7.32 (m, 5*H*), 7.16 (d, *J* = 2.1 Hz, 1*H*), 6.99 (dd, *J* = 8.8 Hz, 2.2 Hz, 1*H*), 5.17 (s, 2*H*); 13C-NMR (DMSO-*d*_6_), δ: 171.65, 162.20, 153.05, 145.72, 136.23, 131.09, 128.48, 128.25, 128.17, 109.28, 106.96, 104.72, 66.14.

#### 3.2.1. General Procedure Used to Synthesize the Carbamates **1**–**32**

4-{[(Benzyloxy)carbonyl]amino}-2-hydroxybenzoic acid (0.6 mmol; 0.2 g) was suspended in dry chlorobenzene (6 mL) at ambient temperature and phosphorus trichloride (0.3 mM, 0.5 eq.), and the corresponding substituted aniline (0.6 mM, 1 eq.) was added dropwise. The reaction mixture was transferred to the microwave reactor, where the synthesis was performed (30 min, 130 °C). Then, the mixture was cooled to 40 °C, and then the solvent was removed to dryness under reduced pressure. The residue was washed with hydrochloride acid and water. The crude product was recrystallized from ethanol.

##### (1) Benzyl[3-hydroxy-4-(phenylcarbamoyl)phenyl]carbamate (**1**)

Yield: 69%; Mp 170–172 °C; HPLC purity: 98.31%; IR (ATR, cm^−1^): 3326, 3033, 1742, 1701, 1635, 1618, 1536, 1498, 1446, 1434, 1328, 1298, 1279, 1219, 1191, 1142, 1097, 1076, 1062, 1029, 967, 850, 763, 750, 692, 598, 564, 508, 464; ^1^H-NMR (DMSO-*d*_6_), δ: 12.14 (s, 1*H*), 10.24 (s, 1*H*), 10.09 (s, 1*H*), 7.93 (d, *J =* 8.3 Hz, 1*H*), 7.67 (d, *J =* 8.3 Hz, 2*H*), 7.45-–7.35 (m, 7*H*), 7.23 (d, *J =* 2.8 Hz, 1*H*), 7.13 (t, *J =* 6.9 Hz, 1*H*), 7.03 (dd, *J =* 8.3 Hz, *J =* 2.8 Hz, 1*H*), 5.18 (s, 2*H*); ^13^C-NMR (DMSO-*d*_6_), δ: 167.10, 160.54, 153.65, 144.68, 138.66, 136.86, 130.22, 129.26, 129.03, 128.77, 128.71, 124.62, 121.60, 111.43, 109.52, 105.88, 66.59; HR-MS: C_21_H_19_O_4_N_2_ [M + H]^+^ calcd 363.1339 *m*/*z*, found 363.1337 *m*/*z.*

##### (2) Benzyl{3-hydroxy-4-[(2-methoxyphenyl)carbamoyl]phenyl}carbamate (**2**)

Yield: 68%; Mp 159–162 °C; HPLC purity: 98.24%; IR (ATR, cm^−1^): 3350, 2966, 1737, 1607, 1578, 1556, 1486, 1376, 1284, 1210, 125,2 1219, 1206, 1189, 1142, 1094, 1052, 1028, 963, 938, 926, 912, 840, 822, 792, 743, 691, 601, 571, 531, 496; ^1^H-NMR (DMSO-*d*_6_), δ: 11.74 (s, 1*H*), 10.66 (s, 1*H*), 10.06 (s, 1*H*), 8.35 (d, *J =*8.2 Hz, 1*H*), 7,91 (d, *J =* 8.2 Hz, 1*H*), 7.45–7.43 (m, 2*H*), 7.42–7.39 (m, 2*H*), 7.37–7.34 (m, 2*H*), 7.08–7.06 (m, 2*H*), 7.01–6.99 (m, 1*H*), 6.95–6.93 (m, 1*H*), 5.17 (s, 2*H*), 3.87 (s, 3*H*); ^13^C-NMR (DMSO-*d*_6_) δ: 163.33; 157.09; 153.13; 148.66; 143.60; 136.38; 131.36; 128.48; 128.19; 128.14; 128.00; 123.68; 120.57; 120.29; 113.04; 110.93; 109.64; 105.20; 65.99; 55.98; HR-MS: C_22_H_21_O_5_N_2_ [M + H]^+^ calcd 393.1445 *m*/*z*, found 393.1442 *m*/*z*.

##### (3) Benzyl{3-hydroxy-4-[(3-methoxyphenyl)carbamoyl]phenyl}carbamate (**3**)

Yield: 48%; Mp 196–198 °C; HPLC purity: 98.13%; IR (ATR, cm^−1^): 3381, 2967, 2881, 1741, 1609, 1583, 1741, 1609, 1583, 1519, 1457, 1434, 1409, 1308, 1291, 1259, 1214, 1200, 1163, 1092, 1052, 967, 872, 842, 763, 749, 715, 697, 685, 599, 577, 492; ^1^H NMR (DMSO-*d*_6_) δ: 12.08 (s, 1*H*), 10.20 (s, 1*H*), 10.08 (s, 1*H*), 7.91 (d, *J =* 8.8 Hz, 1*H*), 7.46–7.34 (m, 6*H*), 7.28–7.21 (m, 3*H*), 7.03 (dd, *J =* 8.8 Hz, *J =* 2.2 Hz, 1*H*), 6.72–6.89 (m, 1*H*), 5.17 (s, 2*H*), 3.76 (s, 3*H*), ^13^C NMR (100 MHz, DMSO-*d*_6_) δ: 166.47, 159.85, 159.48, 153.11, 144.14, 139.36, 136.32, 129.74. 129.50, 128.49, 128.23, 128.16, 113.14, 111.02, 109.55, 109.01, 106.64, 105.34, 66.07, 55.07; HR-MS: C_22_H_21_O_5_N_2_ [M + H]^+^ calcd 393.1440 *m*/*z*, found 393.1442 *m*/*z*.

##### (4) Benzyl{3-hydroxy-4-[(4-methoxyphenyl)carbamoyl]phenyl}carbamate (**4**)

Yield: 65%; Mp 183–186 °C; HPLC purity: 98.84%; IR (ATR, cm^−1^): 3431, 3308, 1740, 1967, 1620, 1572, 1510, 1418, 1365, 1309, 1244, 1199, 1140, 1065, 1033, 960, 845, 829, 762, 736, 729, 695, 680, 533, 520, 462; ^1^H-NMR (DMSO-*d*_6_), δ: 12.34 (s, 1*H*), 10.15 (s, 1*H*), 10.07 (s, 1*H*), 7.93 (d, *J* = 8.9 Hz, 1*H*), 7.57 (d, *J* = 8.9 Hz, 2*H*), 7.45–7.34 (m, 5*H*), 7.20 (s, 1*H*), 7.03 (dd, J = 8.9 Hz, *J* = 1.4 Hz, 1*H*), 6.94 (d, J = 8.9 Hz, 2*H*), 5.18 (s, 2*H*), 3.75 (s, 3*H*); ^13^C NMR (100 MHz, DMSO-*d*_6_) δ: 166.71, 160.49, 155.98, 153.13, 144.10, 136.34, 130.94, 129.26, 128.49, 128.24, 128.17, 122.95, 113.84, 110.45, 108.84, 105.40, 66.07, 55.20; HR-MS: C_22_H_21_O_5_N_2_ [M + H]^+^ calcd 393.1445 *m*/*z*, found 393.1442 *m*/*z*.

##### (5) Benzyl{3-hydroxy-4-[(3-methylphenyl)carbamoyl]phenyl}carbamate (**5**)

Yield: 50%; Mp 167–170 °C; HPLC purity: 98.51%; IR (ATR, cm^−1^): 3326, 3186, 3032, 1702, 1636, 1613, 1570, 1535, 1492, 1433, 1363, 1300, 1244, 1199, 1178, 1136, 1109, 1070, 967, 853, 768, 730, 696, 685, 674, 634, 546, 442; ^1^H-NMR (DMSO-*d*_6_), δ: 12.17 (br s, 1*H*), 10.18 (s, 1*H*), 10.08 (s, 1*H*), 7.94 (d, *J =* 8.9 Hz, 1*H*), 7.51 (s, 1*H*) 7.48–7.47 (m, 1*H*), 7.45–7.43 (m, 2*H*), 7.42–7.39 (m, 2*H*), 7.37–7.34 (m, 1*H*) 7.25–7.23 (m, 2*H*), 7,03 (dd, *J =* 8.9 Hz, *J =* 2.1 Hz, 1*H*), 6.95 (d, *J =* 7.6 Hz, 1*H*), 5.17 (s, 2*H*), 2.31 (s, 3*H*); ^13^C-NMR (DMSO-*d*_6_) δ: 166.49, 160.00, 153.13, 144.14, 138.06, 137.95, 136.33, 129.69, 128.56, 128.49, 128.24, 128.17, 124.79, 121.56, 118.21, 110.90, 109.01, 105.37, 66.07, 21.14; HR-MS: C_22_H_21_O_4_N_2_ [M + H]^+^ calcd 377.1496 *m*/*z*, found 377.1491 *m*/*z*.

##### (6) Benzyl{4-[(2-fluorophenyl)carbamoyl]-3-hydroxyphenyl}carbamate (**6**)

Yield: 68%; Mp 173–176 °C; HPLC purity: 98.48%; IR (ATR, cm^−1^): 3319, 2981, 2970, 2883, 1699, 1645, 1618, 1591, 1566, 1535, 1487, 1458, 1435, 1361, 1338, 1287, 1277, 1244, 1198, 1185, 1153, 1136, 1089, 1064, 1032, 969, 926, 839, 785, 790, 744, 735, 695, 674, 616, 599, 570, 531, 463, 432; ^1^H-NMR (DMSO-*d*_6_), δ: 12.00 (s, 1*H*), 10.54 (d, *J =* 1.4 Hz, 1*H*), 10.11 (s, 1*H*), 8.19 (td, *J =* 7.9 Hz, *J =* 1.4 Hz, 1*H*), 7.92 (d, *J =* 8.2 Hz, 1*H*), 7.45–7.43 (m, 2*H*), 7.42–7.39 (m, 2*H*), 7.37–7.35 (m, 2*H*), 7.31 (ddd, *J =* 11.2 Hz, *J =* 8.1 Hz, *J =* 1.4 Hz, 1*H*), 7.21 (td, *J =* 7.6 Hz, *J =* 1.4 Hz, 1*H*), 7.17–7.15 (m, 1*H*), 7.02 (dd, *J =* 8.9 Hz, *J =* 2.1 Hz, 1*H*), 5.18 (s, 2*H*); ^13^C-NMR (DMSO-*d*_6_) δ: 164.49, 158.08, 153.29 (d, *J =* 244.2 Hz), 153.12, 144.14, 136.33, 130.94, 128.48, 128.22, 128.16, 126.29 (d, *J =* 10.1 Hz), 124.97 (d, *J =* 7.2 Hz), 124.64 (d, *J =* 2.9 Hz), 123.25, 115.25 (d, *J =* 18.8 Hz), 111.70, 109.63, 105.19, 66.05; HR-MS: C_21_H_18_O_4_N_2_F [M + H]^+^ calcd 381.1245 *m*/*z*, found 381.1243 *m*/*z*.

##### (7) Benzyl{4-[(3-fluorophenyl)carbamoyl]-3-hydroxyphenyl}carbamate (**7**)

Yield: 63%; Mp 195–196 °C; HPLC purity: 98.52%; IR (ATR, cm^−1^): 3428, 3326, 3038, 1732, 1618, 1604, 1534, 1492, 1437, 1386, 1329, 1302, 1279, 1253, 1201, 1177, 1154, 1109, 1082, 1061, 1029, 991, 980, 948, 889, 858, 839, 773, 762, 733, 695, 677, 635, 597, 547, 520, 466; ^1^H NMR (DMSO-*d*_6_), δ: 11.92 (s, 1*H*), 10.36 (s, 1*H*), 10.09 (s, 1*H*), 7.89 (d, *J =* 8.8 Hz, 1*H*), 7.69 (dt, *J =* 11.6 Hz, *J =*2.2 Hz, 1*H*), 7.45–7.34 (m, 7*H*), 7.26 (d, *J =* 2.0 Hz, 1*H*), 7.03 (dd, *J =* 8.7 Hz, *J =* 2.1 Hz, 1*H*), 6.97–6.93 (m, 1*H*), 5.17 (s, 2*H*), ^13^C NMR (DMSO-*d*_6_), δ: 166.46, 162.09 (d, *J =* 241.3 Hz), 159.63, 153.13, 144.29, 140.07 (d, *J =* 10.1 Hz), 136.33, 130.33 (d, *J =* 8.7 Hz), 129.98, 128.49, 128.25, 128.18, 116.49 (d, *J =* 2.3 Hz), 111.16, 110.39 (d, *J =* 20.2 Hz), 109.14, 107.56 (d, *J =* 26.0 Hz), 105.35, 66.09; HR-MS: C_21_H_18_O_4_N_2_F [M + H]^+^ calcd 381.1245 *m*/*z*, found 381.1242 *m*/*z*.

##### (8) Benzyl{4-[(4-fluorophenyl)carbamoyl]-3-hydroxyphenyl}carbamate (**8**)

Yield: 59%; Mp 170–173 °C; HPLC purity: 98.49%; IR (ATR, cm^−1^): 3410, 3326, 2988, 2900, 1722, 1613, 1601, 1531, 1508, 1455, 1434, 1406, 1393, 1322,1278, 1258, 1195, 1157, 1093, 1080, 1054, 1028, 976, 892, 866, 826, 775, 762, 732, 694, 667, 635, 587, 511, 466, 444; ^1^H-NMR (DMSO-*d*_6_), δ: 12.12 (s, 1*H*), 10.27 (s, 1*H*), 10.08 (s, 1*H*), 7.91 (d, *J =* 8.9 Hz, 1*H*), 7.69 (dd, *J =* 8.2 Hz, *J =* 4.8 Hz, 2H), 7.45–7.35 (m, 5*H*), 7.22–7.19 (m, 3*H*), 7.03 (d, *J =* 8.2 Hz, 1*H*), 5.17 (s, 2H); ^13^C-NMR (DMSO-*d*_6_) δ: 166.66, 160.12, 158.61 (d, *J =* 241.3 Hz), 153.13, 144.20, 136.33, 134.45 (d, *J =* 2.9 Hz), 129.58, 128.51, 128.25, 128.18, 123.07 (d, *J =* 8.7 Hz), 115.33 (d, *J =* 23.1 Hz), 110.69, 108.99, 105.37, 66.08; HR-MS: C_21_H_18_O_4_N_2_F [M + H]^+^ calcd 381.1245 *m*/*z*, found 381.1242 *m*/*z*.

##### (9) Benzyl{4-[(2-chlorophenyl)carbamoyl]-3-hydroxyphenyl}carbamate (**9**)

Yield: 52%; Mp 156–158 °C; HPLC purity: 98.79%; IR (ATR, cm^−1^): 3419, 2981, 2970, 2883, 1749, 1620, 1596, 1585, 1532, 1458, 1440, 1417, 1382, 1366, 1301, 1278, 1263, 1196, 1158, 1141, 1127, 1109, 1068, 1049, 1036, 980, 950, 865, 849, 834, 766, 751, 736, 693, 681, 658, 600, 570, 548, 548, 843, 468, 462; ^1^H-NMR (DMSO-*d*_6_), δ: 11.98 (s, 1*H*), 10.76 (s, 1*H*), 10.11 (s, 1*H*), 8.39 (dd, *J =* 8.2 Hz, *J =* 1.4 Hz, 1*H*), 7.94 (d, *J =* 8.9 Hz, 1*H*), 7.54 (dd, *J =* 8.2 Hz, *J =* 1.4 Hz, 1*H*), 7.45–7.44 (m, 2*H*), 7.42–7.35 (m, 5*H*), 7.15 (td, *J =* 7.7 Hz, *J =* 1.7 Hz, 1*H*), 7.03 (dd, *J =* 8.9 Hz, *J =* 2.1 Hz, 1*H*), 5.17 (s, 2H); ^13^C-NMR (DMSO-*d*_6_) δ: 163.92, 157.54, 153.12, 144.10, 136.34, 135.45, 131.37, 129.31, 128.48, 128.21, 128.18, 127.79, 124.99, 123.44, 122.86, 112.18, 109.76, 105.15, 66.04; HR-MS: C_21_H_18_O_4_N_2_Cl [M + H]^+^ calcd 397.0950 *m*/*z*, found 397.0949 *m*/*z*.

##### (10) Benzyl{4-[(3-chlorophenyl)carbamoyl]-3-hydroxyphenyl}carbamate (**10**)

Yield: 64%; Mp 231–233 °C; HPLC purity: 98.85%; IR (ATR, cm^−1^): 3403, 2981, 2883, 1739, 1611, 1599, 1545, 1522, 1456, 1428, 1382, 1306, 1246, 1214, 1202, 1142, 1111, 1074, 1053, 964, 912, 888, 849, 794, 778, 767, 748, 696, 679, 600, 578, 565, 541, 491, 462; ^1^H-NMR (DMSO-*d*_6_), δ: 11.92 (s, 1*H*), 10.33 (s, 1*H*), 10.10 (s, 1*H*), 7.90 (s, 1*H*) 7.89 (d, *J =* 8.9 Hz, 1*H*), 7.59 (d, *J =* 8.2 Hz, 1*H*), 7.45–7.34 (m, 6*H*), 7.26 (br.s, 1*H*), 7.18 (d, *J =* 7.6 Hz, 1*H*), 7.03 (d, *J =* 8.2 Hz, 1*H*), 5.17 (s, 2*H*); ^13^C-NMR (DMSO-*d*_6_) δ: 166.47, 159.64, 153.11, 144,28, 139.77, 136.31, 133.00, 130.38, 129.93, 128.49, 128.24, 128.17, 123.64, 120.25, 119.18, 111.08, 109.11, 105.31, 66.08; HR-MS: C_21_H_18_O_4_N_2_Cl [M + H]^+^ calcd 397.0949 *m*/*z*, found 397.0948 *m*/*z*.

##### (11) Benzyl{4-[(4-chlorophenyl)carbamoyl]-3-hydroxyphenyl}carbamate (**11**)

Yield: 54%; Mp 209–212 °C; HPLC purity: 98.87%; IR (ATR, cm^−1^): 3430, 3409, 3315, 2988, 2900, 1743, 1725, 1618, 1572, 1525, 1489, 1461, 1453, 1434, 1420, 1398, 1368, 1311, 1276, 1205, 1090, 1057, 1028, 1010, 985, 963, 905, 892, 871, 831, 825, 819, 762, 734, 706, 694, 683, 657, 574, 543, 513, 506, 443; ^1^H-NMR (DMSO-*d*_6_), δ: 12.0 (s, 1*H*), 10.31 (s, 1*H*), 10.09 (s, 1*H*), 7.89 (d, *J =* 8.9 Hz, 1*H*), 7.73–7.71 (m, 2*H*), 7.45–7.39 (m, 6*H*), 7.37–7.34 (m, 1*H*), 7.24 (d, *J =* 2.1 Hz, 1*H*), 7.03 (dd, *J =* 8.9 Hz, *J =* 2.1 Hz, 1*H*), 5.17 (s, 2*H*); ^13^C-NMR (DMSO-*d*_6_) δ: 166.48, 159.80, 153.11, 144.22, 137.19, 136.31, 129.79, 128.62, 128.49, 128.23, 128.17, 127.65, 112.47, 110.96, 109.04, 105.32, 66.07; HR-MS: C_21_H_18_O_4_N_2_Cl [M + H]^+^ calcd 397.0949 *m*/*z*, found 397.0952 *m*/*z*.

##### (12) Benzyl(3-hydroxy-4-{[2-(trifluoromethyl)phenyl]carbamoyl}phenyl)carbamate (**12**)

Yield: 65%; Mp 173–175 °C; HPLC purity: 98.74%; IR (ATR, cm^−1^): 3367, 2981, 2883, 1716, 1643, 1616, 1594, 1530, 1506, 1458, 1434, 1386, 1309, 1260, 1218, 1180, 1170, 1102, 1057, 961, 870, 819, 783, 765, 731, 693, 658, 634, 574, 545, 462; ^1^H-NMR (DMSO-*d*_6_), δ: 11.99 (s, 1*H*), 10.64 (s, 1*H*), 10.11 (s, 1*H*), 8.19 (d, *J =* 8.2 Hz, 1*H*), 7.92 (d, *J =* 8.9 Hz, 1*H*), 7.75 (d, *J =* 7.6 Hz, 1*H*), 7.70 (t, *J =* 7.9 Hz, 1*H*), 7.45–7.44 (m, 2*H*), 7.42–7.34 (m, 5*H*), 7.03 (dd, *J =* 8.9 Hz, *J =* 1.4 Hz, 1*H*), 5.18 (s, 2*H*); ^13^C-NMR (DMSO-*d*_6_) δ: 164.51, 157.92, 153.12, 144.24, 136.33, 135.63, 133.21, 131.27, 128.48, 128.22, 128.16, 126.10, 126.21–126.13 (m), 124.97, 123.91 (q, *J =* 273.1 Hz), 120.74 (q, *J =* 28.9 Hz), 111.66, 109.69, 105.12, 66.06; HR-MS: C_22_H_18_O_4_N_2_F_3_ [M + H]^+^ calcd 431.1213 *m*/*z*, found 431.1205 *m*/*z*.

##### (13) Benzyl(3-hydroxy-4-{[3-(trifluoromethyl)phenyl]carbamoyl}phenyl)carbamate (**13**)

Yield: 53%; Mp 213–216 °C; HPLC purity: 98.84%; IR (ATR, cm^−1^): 3440, 3320, 1739, 1613, 1584, 1571, 1524, 1452, 1414, 1343, 1325, 1268, 1250, 1238, 1214, 1202, 1185, 1176, 1165, 1111, 1069, 1054, 964, 913, 897, 870, 846, 795, 768, 747, 695, 659, 601, 574, 565, 542, 524, 491, 461; ^1^H-NMR (DMSO-*d*_6_), δ: 11.90 (s, 1*H*), 10.47 (s, 1*H*), 10.11 (s, 1*H*), 8.19 (s, 1*H*), 7.94–7.90 (m, 2*H*), 7.60 (t, *J =* 7.9 Hz, 1*H*), 7.47–7.44 (m, 3*H*), 7.42–7.39 (m, 2*H*), 7.37–7.34 (m, 1*H*), 7.27 (d, *J =* 2.1 Hz, 1*H*), 7.05 (dd, *J =* 8.6 Hz, *J =* 1.7 Hz, 1*H*), 5.18 (s, 2*H*); ^13^C-NMR (DMSO-*d*_6_) δ: 166.71, 159.73, 153.12, 144.37, 139.13, 136.32, 129.94, 129.92, 129.44 (q, *J =* 31.8 Hz), 128.49, 128.25, 128.17, 124.38, 124.12 (q, *J =* 273.1 Hz), 120.24 (q, *J =* 4.3 Hz), 116.90 (q, *J =* 4.3 Hz), 111.04, 109.13, 105.34, 66.09. HR-MS: C_22_H_18_O_4_N_2_F_3_ [M + H]^+^ calcd 431.1213 *m*/*z*, found 431.1206 *m*/*z*.

##### (14) Benzyl(3-hydroxy-4-{[4-(trifluoromethyl)phenyl]carbamoyl}phenyl)carbamate (**14**)

Yield: 61%; Mp 233–235 °C; HPLC purity: 98.81%; IR (ATR, cm^−1^): 3354, 2981, 2970, 2888, 1701, 1637, 1620, 1604, 1589, 1556, 1530, 1501, 1461, 1434, 1411, 1327, 1282, 1237, 1180, 1156, 1113, 1100, 1067, 1014, 964, 855, 841, 823, 770, 761, 695, 678, 665, 622, 587, 510; ^1^H-NMR (DMSO-*d*_6_), δ: 11.88 (s, 1*H*), 10.50 (s, 1*H*), 10.11 (s, 1*H*), 7.93–7.92 (m, 2*H*), 7.91 (d, *J =* 8.9 Hz, 1*H*), 7.72 (d, *J =* 8.9 Hz, 1*H*), 7.45–7.44 (m, 2*H*), 7.42–7.39 (m, 2*H*), 7.37–7.34 (m, 2*H*), 7.28 (s, 1*H*), 7.04 (dd, *J =* 8.9 Hz, *J =* 2.1 Hz, 1*H*), 5.18 (s, 2*H*); ^13^C-NMR (DMSO-*d*_6_) δ: 166.46, 159.45, 153.11, 144.35, 142.01, 136.30, 130.17, 128.48, 128.24, 128.17, 126.16 (q, *J =* 271.7 Hz), 125.99 (q, *J =* 4.3 Hz), 123.79 (q, *J =* 31.8 Hz), 120.54, 111.28, 109.19, 105.29, 66.08; HR-MS: C_22_H_18_O_4_N_2_F_3_ [M + H]^+^ calcd 431.1213 *m*/*z*, found 431.1207 *m*/*z*.

##### (15) Benzyl(3-hydroxy-4-{[2-(trifluoromethoxy)phenyl]carbamoyl}phenyl)carbamate (**15**)

Yield: 49%; Mp 185–187 °C; HPLC purity: 98.27%; IR (cm^−1^): 3524, 3290, 3032, 1707, 1607, 1540, 1521, 1455, 1436, 1414, 1327, 1305, 1240, 1203, 1178, 1166, 1153, 1093, 1057, 964, 927, 858, 753, 734, 692, 630, 579, 551, 461; ^1^H-NMR (DMSO-*d*_6_), δ: 11.98 (s, 1*H*), 10.84 (s, 1*H*), 10.12 (s, 1*H*), 8.49 (dd, *J =* 8.2 Hz, *J =* 1.4 Hz, 1*H*), 7.93 (d, *J =* 8.2 Hz, 1*H*), 7.45–7.39 (m, 7*H*), 7.37–7.34 (m, 1*H*), 7.21 (td, *J =* 7.7 Hz, *J =* 1.7 Hz, 1*H*), 7.04 (dd, *J =* 8.2 Hz, *J =* 2.1 Hz, 1*H*), 5.18 (s, 2*H*); ^13^C-NMR (DMSO-*d*_6_) δ: 163.55, 157.27, 153.14, 144.13, 137.99, 136.33, 131.65, 131.52, 128.49, 128.25, 128.17, 128.12, 124.9, 122.20, 121.30, 120.26 (q, *J =* 257.2 Hz), 112.31, 109.85, 105.17, 66.07; HR-MS: C_22_H_18_O_5_N_2_F_3_ [M + H]^+^ calcd 447.1162 *m*/*z*, found 447.1153 *m*/*z*.

##### (16) Benzyl{4-[(2,3-difluorophenyl)carbamoyl]-3-hydroxyphenyl}carbamate (**16**)

Yield: 52%; Mp 208–210 °C; HPLC purity: 98.46%; IR (ATR, cm^−1^): 3446, 3356, 3029, 1748, 1637, 1618, 1575, 1530, 1476, 1456, 1432, 1405, 1310, 1268, 1219, 1206, 1176, 1149, 1104, 1096, 1063, 1048, 998, 983, 964, 882, 851, 834, 758, 730, 692, 684, 662, 639, 562, 531, 472, 462; ^1^H-NMR (DMSO-*d*_6_) δ: 12.01 (s, 1*H*), 10.64 (s, 1*H*), 10.13 (s, 1*H*), 8.01–7.99 (m, 1*H*), 7.92 (d, *J =* 8.2 Hz, 1*H*), 7.45–7.44 (m, 2*H*), 7.42–7.39 (m, 2*H*), 7.37–7.34 (m, 2*H*), 7.24–7.18 (m, 2*H*), 7.03 (dd, *J =* 8.2 Hz, *J =* 2.1 Hz, 1*H*), 5.18 (s, 2*H*); ^13^C-NMR (DMSO-*d*_6_) δ: 164.50, 158.09, 153.12, 149.79 (dd, *J =* 244.2 Hz, *J =* 10.1 Hz), 144.33, 141.7 (dd, *J =* 245.7 Hz, *J =* 14.5 Hz), 136.31, 131.06, 128.48, 128.23, 128.16, 124.57–124.48 (m), 118.58 (d, *J =* 2.9 Hz), 112.26 (d, *J =* 15.9 Hz), 111.50, 109.70, 105.18, 66.07; HR-MS: C_21_H_17_O_4_N_2_F_2_ [M + H]^+^ calcd 399.1151 *m*/*z*, found 399.1145 *m*/*z*.

##### (17) Benzyl{4-[(2,4-difluorophenyl)carbamoyl]-3-hydroxyphenyl}carbamate (**17**)

Yield: 61%; Mp 218–219 °C; HPLC purity: 98.33%; IR (ATR, cm^−1^): 3325, 3257, 1700, 1651, 1619, 1592, 1573, 1537, 1497, 1455, 1436, 1362, 1346, 1298, 1277, 1244, 1209, 1187, 1142, 1105, 1086, 1066, 1030, 993, 974, 962, 906, 887, 842, 825, 814, 788, 769, 759, 733, 695, 673, 658, 615, 599, 575, 549, 504, 473, 463, 454; ^1^H-NMR (DMSO-*d*_6_), δ: 12.02 (s, 1*H*), 10.45 (s, 1*H*), 10.1 (s, 1*H*), 8.09 (td, *J =* 9.1 Hz, *J =* 6.5 Hz, 1*H*), 7.91 (d, *J =* 8.9 Hz, 1*H*), 7.46–7.44 (m, 2*H*), 7.42–7.34 (m, 4*H*), 7.33 (d, *J =* 1.4 Hz, 1*H*), 7.14–7.10 (m, 1*H*), 7.03 (dd, *J =* 8.9 Hz, *J =* 2.1 Hz, 1*H*), 5.17 (s, 2*H*); ^13^C-NMR (DMSO-*d*_6_), δ: 165.00, 158.54, 158.45 (dd, *J =* 242.8 Hz, *J =* 13.0 Hz), 153.82 (dd, *J =* 247.1 Hz, *J =* 13.0 Hz), 153.14, 144.25, 136.33, 130.69, 128.50, 128.25, 128.18, 124.99 (dd, *J =* 10.1 Hz, *J =* 2.9 Hz), 122.73 (dd, *J =* 11.6 Hz, *J =* 4.3 Hz), 111.37 (d, *J =* 2.9 Hz), 111.23, 109.55, 105.22, 104.11 (dd, *J =* 27.5 Hz, *J =* 24.6 Hz), 66.09; HR-MS: C_21_H_17_O_4_N_2_F_2_ [M + H]^+^ calcd 399.1151 *m*/*z*, found 399.1146 *m*/*z*.

##### (18) Benzyl{4-[(2,5-difluorophenyl)carbamoyl]-3-hydroxyphenyl}carbamate (**18**)

Yield: 65%; Mp 215–216 °C; HPLC purity: 98.32%; IR (ATR, cm^−1^): 3326, 2968, 2880, 2360, 1702, 1643, 1614, 1572, 1540, 1505, 1483, 1436, 1369, 1314, 1287, 1247, 1195, 1170, 1115, 1067, 1030, 1000, 988, 967, 909, 884, 839, 824, 787, 762, 737, 696, 667, 630, 620, 591, 553, 513, 464; ^1^H-NMR (DMSO-*d*_6_), δ: 11.73 (s, 1*H*), 10.44 (s, 1*H*), 10.10 (s, 1*H*), 7.85 (d, *J =* 8.2 Hz, 1*H*), 7.49 (dd, *J =* 9.6 Hz, *J =* 2.7 Hz, 2*H*), 7.46–7.44 (m, 2*H*), 7.42–7.39 (m, 2*H*), 7.37–7.39 (m, 1*H*), 7.28 (d, *J =* 2.1 Hz, 1*H*), 7.03 (dd, *J =* 8.9 Hz, *J =* 2.1 Hz, 1*H*), 6.97 (tt, *J =* 9.3 Hz, *J =* 2.4 Hz, 1*H*), 5.18 (s, 2H); ^13^C-NMR (DMSO-*d*_6_), δ: 166.34, 162.41 (d, *J =* 244.2 Hz), 162.31 (d, *J =* 242.8 Hz), 159.23, 153.12, 144.36, 140.98 (t, *J =* 13.0 Hz), 136.31, 130.18, 128.49, 128.24, 128.18, 111.40, 109.24, 105.29, 103.39 (m, 2C), 98.89 (t, *J =* 26,0 Hz), 66.09; HR-MS: C_21_H_17_O_4_N_2_F_2_ [M + H]^+^ calcd 399.1151 *m*/*z*, found 399.1149 *m*/*z*.

##### (19) Benzyl{4-[(2,6-difluorophenyl)carbamoyl]-3-hydroxyphenyl}carbamate (**19**)

Yield: 69%; Mp 175–178 °C; HPLC purity: 98.48%; IR (ATR, cm^−1^): 3405, 3326, 3035, 1736, 1632, 1615, 1591, 1511, 1497, 1470, 1419, 1372, 1309, 1294, 1270, 1242, 1215, 1198, 1111, 1058, 1012, 990, 963, 915, 848, 826, 776, 760, 741, 721, 700, 650, 628, 558, 528, 509, 483, 455, 444; ^1^H-NMR (DMSO-*d*_6_), δ: 12.09 (s, 1*H*), 10.14 (s, 1*H*), 10.12 (s, 1*H*), 7.95 (d, *J =* 8.9 Hz, 1*H*), 7.46–7.34 (m, 6*H*), 7.27–7.21 (m, 3*H*), 7.06 (d, *J =* 8.2 Hz, 1*H*), 5.19 (s, 2*H*); ^13^C-NMR (DMSO-*d*_6_), δ: 167.20, 160.56, 158.11 (dd, *J =* 249 Hz, *J =* 5.8 Hz), 153.16, 144.73, 136.31, 129.75, 128.52, 128.30, 128.20, 114.05 (t, *J =* 17.3 Hz), 111.95 (dd, *J =* 20.2 Hz, *J =* 4.3 Hz), 109.43, 109.22, 105.35, 66.17; HR-MS: C_21_H_17_O_4_N_2_F_2_ [M + H]^+^ calcd 399.1151 *m*/*z*, found 399.1144 *m*/*z*.

##### (20) Benzyl{4-[(3,5-difluorophenyl)carbamoyl]-3-hydroxyphenyl}carbamate (**20**)

Yield: 57%; Mp 224–225 °C; HPLC purity: 98.51%; IR (ATR, cm^−1^): 3327, 2968, 1708, 1643, 1614, 1573, 1540, 1505, 1483, 1437, 1369, 1314, 1287, 1247, 1195, 1169, 1115, 1067, 1000, 988, 963, 884, 839, 824, 768, 761, 737, 696, 666, 630, 620, 591, 553, 513, 465; ^1^H-NMR (DMSO-*d*_6_), δ: 11.73 (s, 1*H*), 10.44 (s, 1*H*), 10.10 (s, 1*H*), 7.84 (d, *J =* 8.7 Hz, 1*H*), 7.53–7.31 (m, 6*H*), 7.49 (dd, *J =* 9.8 Hz, *J =* 2.2 Hz, 1*H*), 7.28 (d, *J =* 1.9 Hz, 1*H*), 7.03 (dd, *J =* 8.7 Hz, *J =* 2.1 Hz, 1*H*), 6.96 (tt, *J =* 9.3 Hz, *J =* 2.4 Hz, 1*H*), 5.17 (s, 2*H*); ^13^C-NMR (DMSO-*d*_6_), δ: 166.34, 162.35 (dd, *J =* 242.8 Hz, *J =* 15.3 Hz), 159.23, 153.10, 144.35, 140.97 (t, *J =* 13.8 Hz), 136.31, 130.16, 128.48, 128.22, 128.16, 111.38, 109.23, 105.30, 103.58–103.20 (m), 98.88 (t, *J =* 26.3 Hz), 66.09; HR-MS: C_21_H_17_O_4_N_2_F_2_ [M + H]^+^ calcd 399.1151 *m*/*z*, found 399.1147 *m*/*z*.

##### (21) Benzyl{4-[(2,3-dichlorophenyl)carbamoyl]-3-hydroxyphenyl}carbamate (**21**)

Yield: 48%; Mp 219–222 °C; HPLC purity: 98.53%; IR (ATR, cm^−1^): 3412, 3260, 3065, 3029, 1744, 1619, 1585, 1577, 1534, 1519, 1453, 1414, 1367, 1329, 1298, 1204, 1191, 1140, 1114, 1080, 1061, 1045, 1030, 980, 962, 886, 849,829, 778, 762, 722, 703, 694, 684, 590, 565, 555, 554, 549, 500; ^1^H-NMR (DMSO-*d*_6_), δ: 12.05 (s, 1*H*), 10.90 (s, 1*H*), 10.12 (s, 1*H*), 8.41 (dd, *J =* 7.6 Hz, *J =* 2.7 Hz, 1*H*), 7.93 (d, *J =* 8.9 Hz, 1*H*), 7.46–7.43 (m, 2*H*), 7.42–7.38 (m, 5*H*), 7.37–7.34 (m, 1*H*), 7.04 (dd, *J =* 8.2 Hz, *J =* 2.1 Hz, 1*H*), 5.18 (s, 2*H*); ^13^C-NMR (DMSO-*d*_6_), δ: 163.90, 157.47, 153.11, 144.27, 137.46, 136.32, 131.73, 131.52, 128.48, 128.43, 128.20, 128.15, 125.17, 121.69, 121.07, 112.08, 109.86, 105.14, 66.06; HR-MS: C_21_H_17_O_4_N_2_Cl_2_ [M + H]^+^ calcd 431.0560 *m*/*z*, found 431.0558 *m*/*z*.

##### (22) Benzyl{4-[(2,5-dichlorophenyl)carbamoyl]-3-hydroxyphenyl}carbamate (**22**)

Yield: 62%; Mp 173–175 °C; HPLC purity: 98.57%; IR (ATR, cm^−1^): 3259, 3112, 3034, 1713, 1699, 1636, 1619, 1577, 1532, 1435, 1405, 1378, 13001, 1276, 1231, 1196, 1140, 1088, 1058, 987, 916, 865, 795, 773, 741, 695, 684, 585, 535, 457, 442; ^1^H-NMR (DMSO-*d*_6_), δ: 12.04 (s, 1*H*), 10.93 (s, 1*H*), 10.14 (s, 1*H*), 8.61 (d, *J =* 2.7 Hz, 1*H*), 7.93 (d, *J =* 8.2 Hz, 1*H*), 7.58 (d, *J =* 8.2 Hz, 1*H*), 7.45–7.39 (m, 5*H*), 7.37–7.34 (m, 1*H*), 7.21 (dd, *J =* 8.6 Hz, *J =* 2.4 Hz, 1*H*), 7.03 (dd, *J =* 8.9 Hz, *J =* 2.1 Hz, 1*H*), 5.17 (s, 2*H*); ^13^C-NMR (DMSO-*d*_6_) δ: 163.68, 157.21, 153.12, 144.35, 136.75, 136.32, 132.03, 131.71, 130.57, 128.49, 128.21, 128.16, 124.14, 121.22, 121.19, 112.16, 109.97, 105.09, 66.07; HR-MS: C_21_H_17_O_4_N_2_Cl_2_ [M + H]^+^ calcd 431.0560 *m*/*z*, found 431.0559 *m*/*z*.

##### (23) Benzyl{4-[(2,6-dichlorophenyl)carbamoyl]-3-hydroxyphenyl}carbamate (**23**)

Yield: 58%; Mp 186–189 °C; HPLC purity: 98.89%; IR (ATR, cm^−1^): 3306, 3083, 3031, 1718, 1660, 1584, 1522, 1444, 1408, 1375, 1353, 1304, 1277, 1248, 1224, 1203, 1162, 1104, 1053, 963, 928, 839, 801, 765, 727, 689, 667, 621, 615, 597, 574, 542, 512, 495, 453; ^1^H-NMR (DMSO-*d*_6_), δ: 11.73 (s, 1*H*), 10.40 (s, 1*H*), 10.11 (s, 1*H*), 7.84 (d, *J =* 8.9 Hz, 1*H*), 7.83 (m, 2H), 7.45–7.44 (m, 2*H*), 7.42–7.39 (m, 2*H*), 7.37–7.34 (m, 1*H*), 7.33 (t, *J =* 2.1 Hz, 1*H*), 7.28 (d, *J =* 2.1 Hz, 1*H*), 7.03 (dd, *J =* 8.2 Hz, *J =* 2.1 Hz, 1*H*), 5.17 (s, 2H); ^13^C-NMR (DMSO-*d*_6_) δ: 166.42, 159.34, 153.11, 144.40, 140.80, 136.30, 134.00, 130.15, 128.50, 128.25, 128.18, 123.00, 118.74, 111.28, 109.22, 105.27, 66.10; HR-MS: C_21_H_17_O_4_N_2_Cl_2_ [M + H]^+^ calcd 431.0560 *m*/*z*, found 431.0561 *m*/*z*.

##### (24) Benzyl{4-[(3,4-dichlorophenyl)carbamoyl]-3-hydroxyphenyl}carbamate (**24**)

Yield: 54%; Mp 255–257 °C; HPLC purity: 98.47%; IR (ATR, cm^−1^): 3407, 3308, 2961, 1739, 1615, 1594, 1520, 1476, 1448, 1414, 1380, 1303, 1277, 1251, 1214, 1199, 1136, 1127, 1104, 1053, 1025, 973, 963, 875, 849, 813, 794, 767, 747, 715, 697, 688, 600, 580, 567, 493, 460, 439; ^1^H-NMR (DMSO-*d*_6_), δ: 11.83 (s, 1*H*); 10.39 (s, 1*H*); 10.10 (s, 1*H*); 8.10 (d, *J =* 2.7 Hz, 1*H*); 7.86 (d, *J =* 8.2 Hz, 1*H*); 7.66 (dd, *J =* 8.9 Hz; *J =* 2.7 Hz, 1*H*); 7.61 (d, *J =* 8.9 Hz, 1*H*); 7.45–7.44 (m, 2*H*); 7.41–7.39 (m, 2*H*); 7.37–7.34 (m, 1*H*); 7.26 (d, *J =* 1.4 Hz, 1*H*); 7.03 (dd, *J =* 8.9 Hz, *J =* 2.1 Hz, 1*H*); 5.17 (s, 2*H*); ^13^C-NMR (DMSO-*d*_6_) δ: 166.42, 159.50, 153.10, 144.35, 138.49, 136.30, 130.92, 130.58, 130.00, 128.49, 128.24, 128.17, 125.36, 121.92, 120.74, 111.12, 109.16, 105.29, 66.08; HR-MS: C_21_H_17_O_4_N_2_Cl_2_ [M + H]^+^ calcd 431.0560 *m*/*z*, found 431.0558 *m*/*z*.

##### (25) Benzyl{4-[(3,5-dichlorophenyl)carbamoyl]-3-hydroxyphenyl}carbamate (**25**)

Yield: 66%; Mp 183–185 °C; HPLC purity: 98.59%; IR (ATR, cm^−1^): 3406, 3331, 2980, 2970, 2883, 1708, 1671, 1648, 1615, 1582, 1532, 1446, 1432, 1532, 1446, 1432, 1415, 1312, 1284, 1241, 1193, 1152, 1132, 1112, 1068, 1029, 962, 932, 861, 832, 796, 765, 737, 694, 665, 643, 610, 566, 545, 494; ^1^H-NMR (DMSO-*d*_6_), δ: 11.73 (s, 1*H*), 10.40 (s, 1*H*), 10.11 (s, 1*H*), 7.84 (d, *J =* 8.9 Hz, 1*H*), 7.83 (d, *J =* 2.1 Hz, 2*H*), 7.45–7.44 (m, 2*H*), 7.42–7.39 (m, 2*H*), 7.37–7.34 (m, 1*H*), 7.33 (t, *J =* 2.1 Hz, 1*H*), 7.28 (d, *J =* 2.1 Hz, 1*H*), 7.03 (dd, *J =* 8.2 Hz, *J =* 2.1 Hz, 1*H*), 5.17 (s, 2*H*); ^13^C-NMR (DMSO-*d*_6_), δ: 166.41, 159.34, 153.10, 144.40, 140.80, 136.29, 133.99, 130.14, 128.48, 128.24, 128.17, 122.99, 118.72, 111.27, 109.21, 105.27, 66.09; HR-MS: C_21_H_17_O_4_N_2_Cl_2_ [M + H]^+^ calcd 431.0560 *m*/*z*, found 431.0564 *m*/*z*.

##### (26) Benzyl{4-[(2,4-dibromophenyl)carbamoyl]-3-hydroxyphenyl}carbamate (**26**)

Yield: 52%; Mp 199–200 °C; HPLC purity: 98.73%; IR (ATR, cm^−1^): 3415, 3090, 1741, 1693, 1630 1618, 1591, 1576, 1521, 1457, 1434, 1417,01, 1373, 1309, 1294, 1238, 1210, 1200, 1150, 1132, 1107, 1081, 1058, 1035, 959, 861, 832, 764, 737, 696, 677, 567, 549, 503, 462; ^1^H-NMR (DMSO-*d*_6_), δ: 12.0 (s, 1*H*), 10.70 (s, 1*H*), 10.10 (s, 1*H*), 8.32 (d, *J =* 8.8 Hz, 1*H*), 7.94 (d, *J =* 2.1 Hz, 1*H*), 7.92 (dd, *J =* 8.9 Hz, *J =* 2.1 Hz, 1*H*), 7.61 (dd, *J =* 8.9 Hz, *J =* 2.1 Hz, 1*H*), 7.45–7.43 (m, 2*H*), 7.41–7.39 (m, 3*H*), 7.36–7.34 (m, 1*H*), 7.03 (dd, *J =* 8.9 Hz, *J =* 2.1 Hz, 1*H*), 5.17 (s, 2*H*); ^13^C-NMR (DMSO-*d*_6_), δ: 163.92, 157.54, 153.11, 144.24, 136.34,136.32, 134.33, 131.45, 131.17, 128.48, 128.20, 128.15, 124.48, 116.00, 114.98, 112.00, 109.61, 105.15, 66.05; HR-MS: C21 H_17_O_4_N_2_Br_2_ [M + H]^+^ calcd 518.9549 *m*/*z*, found 518.9548 *m*/*z*.

##### (27) Benzyl{4-[(3,5-di(trifluoromethyl))carbamoyl]-3-hydroxyphenyl}carbamate (**27**)

Yield: 53%; Mp 189–191 °C; HPLC purity: 98.91%; IR (ATR, cm^−1^): 3327, 2981, 2970, 2883, 1752, 1719, 1643, 1615, 1588, 1535, 1473, 1446, 1431, 1381, 1276, 1224, 1166, 1124, 1166, 1124, 1094, 1058, 996, 937, 884, 866, 840, 761, 734, 670, 683, 613, 605, 541; ^1^H-NMR (DMSO-*d*_6_), δ: 11.67 (s, 1*H*), 10.68 (s, 1*H*), 10.13 (s, 1*H*), 8.45 (s, 2*H*), 7.87 (d, *J =* 8.2 Hz, 1*H*), 7.81 (s, 1*H*), 7.45–7.44 (m, 2*H*), 7.42–7.40 (m, 2*H*), 7.37–7.35 (m, 1*H*), 7.30 (d, *J =* 1.4 Hz, 1*H*), 7.04 (dd, *J =* 8.2 Hz, *J =* 2.1 Hz, 1*H*), 5.18 (s, 2*H*); ^13^C-NMR (DMSO-*d**_6_***) δ: 166.75, 159.43, 153.11, 144.54, 140.42, 136.29, 130.64 (q, *J =* 33.2 Hz), 130.19, 128.49, 128.25, 128.18, 133.26, (q, *J =* 273.1 Hz), 120.43 (q, *J =* 4.3 Hz), 116.55 (m), 111.17, 109.24, 105.26, 66.11; HR-MS: C_23_H_17_O_4_N_2_F_6_ [M + H]^+^ calcd 499.1087 *m*/*z*, found 499.1082 *m*/*z*.

##### (28) Benzyl{3-hydroxy-4-[(2,4,6-trifluorophenyl)carbamoyl]phenyl}carbamate (**28**)

Yield: 68%; Mp 203–206 °C; HPLC purity: 98.17%; IR (ATR, cm^−1^): 3443, 3363, 1739, 1651, 1606, 1508, 1466, 1448, 1416, 1378, 1363, 1336, 1310, 1252, 1211, 1188, 1172, 1117, 1085, 1052, 1039, 1030, 997, 979, 900, 853, 833, 825, 801, 756, 736, 711, 699, 691, 658, 636, 629, 608, 544, 512, 497; ^1^H-NMR (DMSO-*d*_6_), δ: 11.97 (s, 1*H*), 10.13 (s, 1*H*), 10.03 (s, 1H), 7.90 (d, *J =* 8.9 Hz, 1*H*), 7.45–7.43 (m, 2*H*), 7.42–7.39 (m, 2*H*), 7.37–7.33 (m, 3*H*), 7.25 (d, *J =* 2.1 Hz, 1*H*), 7.03 (dd, *J =* 8.9 Hz, *J =* 2.1 Hz, 1*H*), 5.18 (s, 2H); ^13^C-NMR (DMSO-*d*_6_), δ: 167.10, 160.33, 160.29 (dt, *J =* 245.7 Hz, *J =* 14.5 Hz), 158.23 (ddd, *J =* 250 Hz, *J =* 15.9 Hz, *J =* 7.2 Hz), 153.11, 144.70, 136.29, 129.80, 128.50, 128.27, 128.18, 111.1 (td, *J =* 17.3 Hz, *J =* 4.3 Hz), 109.41, 109.0, 105.26, 100.98 (m), 66.13; HR-MS: C_21_H_16_O_4_N_2_F_3_ [M + H]^+^ calcd 417.1057 *m*/*z*, found 417.1050 *m*/*z*.

##### (29) Benzyl{3-hydroxy-4-[(3,4,5-trifluorophenyl)carbamoyl]phenyl}carbamate (**29**)

Yield: 80%; Mp 206–207 °C; HPLC purity: 98.22%; IR (ATR, cm^−1^): 3429, 3334, 3040, 1726, 1616, 1543, 1527, 1438, 1393, 1313, 1285, 1237, 1208, 1182, 1135, 1044, 991, 964, 894, 873, 850, 834, 796, 763, 741, 697, 663, 620, 566, 513, 466, ^1^H-NMR (DMSO-*d*_6_), δ: 11.72 (s, 1*H*), 10.40 (s, 1*H*), 10.10 (s, 1*H*), 7.83 (d, *J =* 8.9 Hz, 1*H*), 7.73–7.75 (m, 2*H*), 7.46–7.43 (m, 2*H*), 7.42–7.39 (m, 2*H*), 7.36–7.34 (m, 1*H*), 7.28 (d, *J =* 2.1 Hz, 1*H*), 7.03 (dd, *J =* 8.2 Hz, *J =* 2.1 Hz, 1*H*), 5.18 (s, 2*H*); ^13^C-NMR (DMSO-*d*_6_), δ: 166.30, 159.24, 153.11, 149.97 (ddd, *J =* 244.2 Hz, *J =* 10.1 Hz, *J =* 5.8 Hz), 144.38, 136.31, 135.01 (dt, *J =* 245.7 Hz, *J =* 15.9 Hz), 134.76 (td, *J =* 11.6 Hz, *J =* 4.3 Hz), 130.10, 128.49, 128.23, 128.17, 111.24, 109.23, 105.27, 104.98 (dd, *J =* 20.2 Hz, *J =* 5.8 Hz), 66.09; HR-MS: C_21_H_16_O_4_N_2_F_3_ [M + H]^+^ calcd 417.1057 *m*/*z*, found 417.1054 *m*/*z*.

##### (30) Benzyl{4-[(2,4,5-trichlorophenyl)carbamoyl]phenyl}carbamate (**30**)

Yield: 62%; Mp: 222–225 °C; HPLC purity: 98.85%; IR (ATR, cm^−1^): 3268, 3119, 1710, 1696, 1619, 1594, 1574, 1519, 1453, 1434, 1360, 1300, 1275, 1235, 1196, 1138, 1071, 1052, 1028, 967, 914, 890, 876, 867, 852, 823, 791, 776, 741, 717, 697, 682, 649, 597, 581, 561, 543, 517, 497; ^1^H-NMR (DMSO-*d*_6_), δ: 12.06 (s, 1*H*), 10.95 (s, 1*H*), 10.13 (s, 1*H*), 8.79 (s, 1*H*), 7.92–7.90 (m, 2*H*), 7.45–7.38 (m, 5*H*), 7.36–7.33 (m, 1*H*), 7.03 (dd, *J =* 8.9 Hz, *J =* 2.1 Hz, 1H), 5.17 (s, 2H); ^13^C-NMR (DMSO-*d*_6_), δ: 163.62, 157.18, 153.10, 144.43, 136.33, 135.67, 131.71, 130.24, 128.47, 128.20, 128.15, 125.37, 122.18, 121.89, 112.03, 110.02, 105.06, 66.08; HR-MS: C_21_H_16_O_4_N_2_Cl_3_ [M + H]^+^ calcd 465.0170 *m*/*z*, found 465.0172 *m*/*z*.

##### (31) Benzyl{3-hydroxy-4-[(2,4,6-trichlorophenyl)carbamoyl]phenyl}carbamate (**31**)

Yield: 54%; Mp 254–256 °C; HPLC purity: 98.81%; IR (ATR, cm^−1^): 3311, 3266, 1711, 1643, 1599, 1507, 1463, 1414, 1372, 1312, 1235, 1188, 1098, 1072, 1055, 967, 915, 853, 818, 803, 771, 755, 739, 693, 654, 561, 530, 458; ^1^H-NMR (DMSO-*d*_6_), δ: 12.05 (s, 1*H*), 10.33 (s, 1*H*), 10.13 (s, 1*H*), 7.94 (d, *J =* 8.9 Hz, 1*H*), 7.82 (s, 2*H*), 7.46–7.44 (m, 2*H*), 7.42–7.39 (m, 2*H*), 7.37–7.34 (1*H*), 7.25 (d, *J =* 1.4 Hz, 1*H*), 7.04 (dd, *J =* 8.6 Hz, *J =* 1.7 Hz, 1*H*), 5.18 (s, 2*H*); ^13^C-NMR (DMSO-*d*_6_), δ: 166.98, 160.63, 153.12, 144.78, 136.29, 134.78, 132.75, 132.09, 129.70, 128.50 128.33, 128.28 128.19, 109.29, 109.21, 105.35, 66.15; HR-MS: C_21_H_16_O_4_N_2_Cl_3_ [M + H]^+^ calcd 465.0170 *m*/*z*, found 465.0170 *m*/*z*.

##### (32) Benzyl{3-hydroxy-4-[(2,4,6-tribromophenyl)carbamoyl]phenyl}carbamate (**32**)

Yield: 60%; Mp 244–245 °C; HPLC purity: 98.78%; IR (ATR, cm^−1^): 3272, 1711, 1643, 1600, 1540, 1506, 1456, 1413, 1370, 1311, 1234, 1189,49, 1054, 846, 770, 739, 693, 650, 570, 548, 522, 456; ^1^H-NMR (DMSO-*d*_6_), δ: 12.12 (s, 1*H*), 10.36 (s, 1*H*), 10.11 (s, 1*H*), 8.07 (s, 2*H*), 7.94 (d, *J =* 8.9 Hz, 1*H*), 7.46–7.44 (m, 2*H*), 7.42–7.39 (m, 2*H*), 4.37–7.34 (m, 1*H*), 7.23 (d, *J =* 2.1 Hz, 1*H*), 7.04 (dd, *J =* 8.6 Hz, *J =* 1.7 Hz, 1*H*), 5.18 (s, 2*H*); ^13^C-NMR (DMSO-*d*_6_), δ: 167.04, 160.92, 153.09, 144.76, 136.27, 135.14, 134.33, 129.45, 128.49, 128.28, 128.18, 125.37, 121.37, 109.12, 109.10, 105.34, 66.14; HR-MS: C_21_H_16_O_4_N_2_Br_3_ [M + H]^+^ calcd 596.8654 *m*/*z*, found 596.8652 *m*/*z*.

### 3.3. Determination of Lipophilicity by HPLC

The HPLC separation system Agilent 1200 series equipped with a DAD SL (Agilent Technologies) was used. A chromatographic column Symmetry^®^ C18 5 μm, 4.6 × 250 mm, Part No. W21751W016 (Waters Corp., Milford, MA, USA) was applied. The HPLC separation process was monitored by the ChemStation for LC 3D chromatography software (Agilent Technologies). Isocratic elution by a mixture of MeOH p.a. (72%) and H_2_O-HPLC Mili-Q grade (28%) as a mobile phase was used for the determination of the capacity factor *k*. The total flow of the column was 1.0 mL/min, injection 20 μL, column temperature 40 °C, and sample temperature 10 °C. The detection wavelength of 210 nm was chosen. A KI methanolic solution was used for the determination of the dead times (*t_d_*). Retention times (*t_r_*) were measured in minutes. The capacity factors *k* were calculated according to the formula *k* = (*t_r_* − *t_d_*)/*t_d_*, where *t_R_* is the retention time of the solute and *t_d_* is the dead time obtained using an unretained analyte. Each experiment was repeated three times. The log *k* values of individual compounds are shown in Table 1.

### 3.4. Evaluating In Vitro AChE and BChE-Inhibition Potencies

The ability of all the prepared compounds to inhibit AChE from electric eel (*Electrophorus electricus*) and BChE from equine serum (both purchased from Sigma, St. Louis, MO, USA) was determined in vitro using a modified Ellman’s method, as described in detail elsewhere [47]. This is a simple, rapid, and direct method to determine the SH and -S-S- group content in proteins. The enzyme activity is measured indirectly by quantifying the concentration of the 5-thio-2-nitrobenzoic acid ion formed in the reaction between the thiol reagent 5,5’-dithiobis-2nitrobenzoic acid (DTNB) and thiocholine, which is a product of acetylthiocholine hydrolysis catalyzed by cholinesterases [48,49,50]. The effectiveness of the tested inhibitors is expressed as the IC_50_ values.

The enzyme activity in the final reaction mixture (2 mL) was 0.2 U/mL, the concentration of the substrate (acetylthiocholine or butyrythiocholine) was 40 µM, and the concentration of DTNB was 0.1 mM for all reactions. All reactions were carried out at 25 °C in the presence of phosphate-buffered saline (0.1 M, pH 7.4) in a glass cuvette with a 1 cm optical path. All the studied inhibitors were dissolved in DMSO (concentration 0.01 M) and then diluted in demineralized water as necessary. For all tested compounds and standards, rivastigmine and galanthamine in at least four different concentrations of inhibitor in the final reaction mixture were used. All measurements were carried out in triplicate, and the average values of the reaction rate (*v*_0_—reaction in the absence of the inhibitor, *v_i_*—reaction in the presence of the inhibitor) were used for construction of the dependence *v*_0_/*v_i_* vs. concentration of the inhibitor. The IC_50_ values were calculated from an obtained equation of the regression curve for y = 2 (resulting from the definition of IC_50_) [14,15,25]. The results are presented in Table 1.

### 3.5. In Vitro Viability Assay

Human monocytic leukemia THP-1 cells were used for in vitro antiproliferative assays. Cells were obtained from the European Collection of Cell Cultures (ECACC, Salisbury, UK) and routinely cultured in Roswell Park Memorial Institute RPMI 1640 medium supplemented with 10% fetal bovine serum (FBS), 2% l-glutamine, 1% penicillin and streptomycin (all from Sigma-Aldrich) at 37 °C with 5% CO_2_. Cells were passaged twice a week. The effect of the compounds on cell viability was determined using a Cell Counting Kit-8 (CCK-8, 2-(2-methoxy-4-nitrophenyl)-3-(4-nitrophenyl)-5-(2,4-disulfophenyl)-2H-tetrazolium, monosodium salt, Sigma) according to the manufacturer’s instructions. The compounds were dissolved in DMSO and added to the cell suspension in the RPMI 1640 medium without FBS (5 × 10^5^ cells/mL). The maximum concentration of DMSO (Sigma) in the assays never exceeded 0.1% (*v*/*v*). Subsequently, the cells were incubated at 37 °C with 5% CO_2_ for 24 h. After this incubation period, the relative cell viability (the ratio between cells treated with compounds and cells treated only with DMSO) was determined by CCK-8 kit.

### 3.6. Model Building and Molecular Modeling

CACTVS/csed and CORINA editors were applied to generate molecule structural model with its 3D geometry. The data format conversion was performed using an OpenBabel (inter)change file format converter. The Sybyl-X 2.0/Certara package installed on a DELL workstation with Ubuntu 20.10 operating system was engaged in order to conduct the molecular modeling simulations. The MAXMIN2 module implemented in Sybyl-X was used to initially optimize the compound spatial geometry with the standard Tripos force field (POWELL conjugate gradient algorithm) with a 0.01 kcal/mol energy gradient convergence criterion. The electrostatic potential values were calculated using Gasteiger–Hückel method. One 18-ordered atom trial alignment was produced on the most active compound **2** (active analogue approach) with an FIT procedure to cover the entire bonding topology in the maximal common structure (MCS). SONNIA software was employed to simulate 10 × 10 to 30 × 30 self-organizing maps (SOMs) with a winning distance in the range from 0.2 to 2.0 in CoMSA analysis. Spatial coordinates of the molecular surfaces and the corresponding potential values were used as input to Kohonen SOM network to generate a 2D map of the electrostatic potential (MEP) for the set of superimposed molecules. The produced maps were reshaped into a 100- to 900-element vector subjected to the PLS method implemented in the MATLAB environment.

### 3.7. Principal Component and Hierarchical Custering Analysis

The human-friendly 2D/3D illustrations of the compound distribution in the experimental-based (FCS) and virtual-derived (VCS) molecular space might be displayed by Principal Component Analysis (PCA). PCA is a linear projection method that can be engaged to model multidimensional data with a relatively small number of so-called principal components (scores and loadings) produced to maximize the description of variance within the input data. The PCA model with *f* principal components for a data matrix *X* can be calculated as follows in Equation (1):

*X* = *TP^T^* + *E* (1)

where *X* is a data matrix with *m* objects and *n* variables, *T* is the score matrix with dimensions (*m* × *f*), *P^T^* is a transposed matrix of loadings with dimensions (*f* × *n*), and *E* is a matrix of the residual variance (*m* × *n*) not explained by the first *f* principal components. On the whole, the first few principal components (PCs) frequently describe sufficiently data variance and reveal the groups of objects.

Hierarchical Clustering Analysis (HCA) enables us to examine the (dis)similarities between objects in the variable space. Hence, the similarity measure as well as the way of resulting sub-clusters linkage should be specified a priori. The produced findings are illustrated as a dendogram, where the OX axis presents the indices of the clustered objects, while the OY one corresponds to the linkage distances between two objects linked, respectively. Moreover, the visualization method can be applied to the empirical data sorted according to the order of objects with the generation of the color-coded variable maps. A mutual interpretation of objects sorted with the Ward linkage method and the color-coded variable maps facilitate the (dis)similarity assessment of objects in terms of the input parameters.

### 3.8. Theoretical Lipophilicity Evaluation

A number of freely/commercially available in silico estimators can be employed to calculate the theoretical partition coefficients (clogP) as follows:

AlogPS—procedure proposed by Tetko et al. based on atom-type electrotopological-state (E-state) indices and neural networks (NN);

milogP—method invented by Molinspiration for practical logP calculations of almost all organic molecules as a sum of fragment-based contributions and correction factors;

ClogP—fragment-based procedure for predicting lipophilicity based on structure-dependent correction values retrieved from Hansch and Leo’s database that is implemented in Sybyl/Centara software;

HyperChem logP—an atom-additive method that approaches lipophilicity using the individual atomic contribution based on procedure proposed by Ghose, Prichett, and Crippen;

MarvinSketch logP—the overall lipophilicity of a molecule is composed of the contributing values of its atom types that were redefined to accommodate electron delocalization and contributions of ionic forms;

ChemSketch logP—a comprehensive fragment-based algorithm with high-quality models retrieved using the empirical data. Well-characterized logP contributions have been compiled for atoms, structural fragments, and intramolecular interactions provided for more than 12 × 10^3^ experimental logP values;

Dragon AlogP—the statistical estimators of the Ghose-Crippen-Viswanadhan model were calculated on the basis of known experimental logP for the training set of 8364 compounds. The overall estimation of the lipophilic atomic-based constant is evaluated with the contribution of 115 atom types;

Dragon MlogP—the theoretical partition coefficient includes VdW volume and Moriguchi polar parameters as correction factors. A regression MlogP model is based on 13 structural parameters, which were evaluated on the training group of 1230 organic molecules;

Kowwin—estimates the log octanol–water partition coefficient of chemicals using the atom/fragment contribution approach;

XlogP3—an atom-additive procedure with well-defined correction factors that employs an optimized atom typing approach calibrated on a big training set;

OSIRIS clogP—in-house approach based on the cumulative sum of atom contributions calculated for more than 5000 compounds with experimentally determined logP values as training set. Predicting engine distinguishes 369 atom types;

ChemBio clogP—the algorithms for prediction of partition coefficient based on a training set of compound provides coverage for a broad chemical space;

Percepta clogP—based on >12 × 10^3^ of experimental logP values with the algorithm that uses the principal of isolating carbons.

The redundant descriptors of QSAR/QSPR investigations were selected and eradicated using the modified version the uninformative variable elimination (UVE–PLS) method, namely iterative variable elimination (IVE–PLS). Briefly, the entire algorithm is composed of the following stages: (1) standard PLS analysis with leave-one-out crossvalidation LOO–CV to evaluate the performance of the PLS model; (2) elimination of the matrix column with the lowest *abs(mean(b)/std(b))* value; (3) standard PLS analysis of the new matrix without the column eliminated in (2); (3) iterative repetition of (**1**)–(**3**) to maximize $q_{cv}^{2}$ value.

### 3.9. Similarity-Based Activity Landscape Index

The numerical profiling of similarity-related structure–activity landscape index (SALI) can be quantitatively expressed according to the following Equation (2)

(2) $$SALI_{x,y}=\frac{\left|A_{x}-A_{y}\right|}{1-sim\left(x,y\right)}$$

where $A_{x}$ and $A_{y}$ are the activity profiles for the x-th and y-th molecule and *sim*(*x,y*) is the pair-wise similarity measure. The Tanimoto coefficient was engaged for the fingerprint-based similarity estimation, where the structural pair-wise molecular relatedness is calculated as follows; in Equation (3):

(3) $$T\left(x,y\right)=\frac{n_{xy}}{\left(n_{x}+n_{y}-n_{xy}\right)}$$

where $n_{xy}$ is the number of bits set into 1 shared in the fingerprint of the molecule *x* and *y*, $n_{x}$ is the number of bits set into 1 in the molecule *x*, $n_{y}$ is the number of bits set into 1 in the molecule *y*, respectively.

### 3.10. Ligand/Structure-Based Analysis

The selection-driven surface analysis of a structural space can be performed using the set of descriptors that grab information about the spatial pharmacophoric pattern. In practice, some machine learning methods conjugated with weighting and selecting procedures were engaged to indicate the minimal ensemble of pharmacophoric features that are potentially important in description of guest-host interactions. The comparative molecular surface analysis (CoMSA) was applied in order to meaningfully compare the shape and charges variations generated on the molecular surface of the receptor and ligand, respectively. Briefly speaking, a single layer of neurons is arranged in a 2D plane with well-defined topology to produce self-organized maps (SOMs). The spatially closed objects are located in the proximal neurons of the square map in the process of SOMs adaptation to the input data. In consequence, a 2D image of the property space is produced, where structurally related molecules are placed in neighboring neurons. The electrostatic/steric features that are potentially important in the ligand–receptor recognition and complementarity phenomena can be specified using the iterative variable selection approaches. The backward column eradication was recurrently repeated until the optimal number of variables included within the model was accomplished—the moment that the $q_{cv}^{2}$ deterioration specified the set of potentially relevant columns. The cumulative sum of the common columns for all of the investigated BChE models was calculated and normalized to a range of (0,1).

The crystallographic structure of butyrylcholinesterase determined using X-ray diffraction at 2.6 Å resolution co-crystalized with a rivastigmine analogue (liganded state) was downloaded from the PDB repository (PDB code: 6eyf). Apart from the RIV-based analogue ([3-[(1~{*R*})-1-(dimethylamino)ethyl]-4-oxidanyl-phenyl]~{*N*}-ethyl- ~{*N*}-methylcarbamate), all remaining heteroatoms (including water molecules) were eradicated prior to docking in the AutoDock Vina programe. Initially, the ligand/enzyme structures were prepared in the pdbqt file format with the calculated Gasteiger charges. The grid box was centered on the central atom of the RIV analogue (BY2). In AutoDock Vina, docking simulations in different poses (default nine) were generated progressively from a single conformer (an energy-minimized molecule). Then, the resulting molecular conformations and orientations with the preferred torsion angles and the rotatable bonds were evaluated by the united-atom (UA) scoring function. VMD, PyMol, Schrödinger Maestro graphical viewers, and Protein–Ligand Interaction Profiler (PLIP) were employed to illustrate the foreseen 2D/3D binding modes, respectively.

Sybyl-X 2.0/Certara modeling software was used to conduct the dynamic simulations of the ligand–enzyme systems. The host–guest structures were analyzed and fixed by the Prepare Protein Structure module. The Berendsen algorithm was used in MD simulations on the NVT ensemble in order to control temperature. The initial atom velocities were assigned at 293K; however, the system was then heated and the corresponding trajectories were determined at the temperature of 300K. The total length of MDs was arranged to 20 ns with 10 fs time step, respectively. The RedMDStream 1.0 program with coarse-grained molecular dynamics models was engaged for the macromolecular simulations in the same conditions.

## 4. Conclusions

In summary:

1. A library of novel 4-{[(benzyloxy)carbonyl]amino}-2-hydroxybenzoic acid amides was designed and synthesized as potential acetyl- and butyrylcholinesterase inhibitors; hence, the in vitro inhibitory profile and selectivity index were determined. Benzyl(3-hydroxy-4-{[2-(trifluoromethoxy)phenyl]carbamoyl}phenyl)carbamate (**15**) showed the best AChE inhibition (IC_50_ = 36.05 µM) and benzyl{3-hydroxy-4-[(2-methoxyphenyl)carbamoyl]phenyl}carbamate (**2**) demonstrated the best BChE inhibiting activity (IC_50_ = 22.23 µM) with the highest selectivity for BChE (SI = 2.26).
2. The distance-oriented property evaluation was conducted using the Principal Component Analysis (PCA) and Hierarchical Clustering Analysis (HCA), respectively. The dissimilarities of molecules **12**–**14**, **27** with trifluoromethyl substituent(s) (-CF_3_) and compound **15** with trifluromethoxy group (-OCF_3_) from the remaining ones was observed. Noticeably, objects **2**–**4** that are methoxy-based (-OCH_3_) positional isomers and molecule **5** with methyl substituent (-CH_3_) were also clustered together.
3. The distribution of the Tanimoto coefficients was analyzed for the triangular T_32×32_ matrix. Interestingly, the potent molecules **2** and **15** are marked as fairly similar (T = 0.93). In fact, molecule **15** is characterized by low values of SALI indexes with the remaining compounds in the dataset, while the most potent molecule **2** is accompanied by gray pots of the inactive molecules **3**, **4** (positional isomers), and **1** (unsubstituted analogue), respectively. Noticeably, three pairs of fairy similar fluorine/bromine-containing compounds (**17** vs. **19** and **28**, **26** vs. **32**) can potentially form the activity cliff that is manifested formally by high SALI numerical values.
4. The preferential selection of the active molecules (**2** and **6**) and inactive ones (**3**, **13**, **23**, and **26**) that are basically *ortho*- and *meta*-substituted analogues resulted in the generation of the robust models with the acceptable predictive power for the test set.
5. The molecular docking approach was engaged for the most potent AChE/BChE inhibitors in order to get comprehensive knowledge of the binding mode. The hydrophobic interactions were overwhelmingly generated with Gln119, Asp70, Pro285, Thr120, and Trp82 aminoacid residues, while the HB-donor ones were dominated with Thr120. π-stacking interactions were specified with Trp82 aminoacid residue of chain A as well. **6.** The stability of some fused ligand–enzyme systems were evaluated using the molecular dynamic simulations. The trifluoromethoxy substituent of molecule **15** is characterized by lower spatial flexibility compared to methoxy group of compound **2**. Obviously, –OCF_3_ is considerably heavier and can generate greater steric hindrance in comparison to –OCH_3_, as the most electronegative element fluorine can interact with the surrounding aminoacid residues, which stiffens the ligand–enzyme system.

## Figures and Tables

**Table 1:** Structure of ring-substituted benzyl[4-(arylcarbamoyl)phenyl-3-hydroxy]carbamates 1–32, experimentally determined log *k* and in vitro acetyl- and butyrylcholinesterase (AChE and BChE) inhibitory concentration (IC_50_) in comparison with starting 4-{[(benzyloxy)carbonyl]amino}-2-hydroxybenzoic acid (CbzPAS) and standards rivastigmine (RIV) and galanthamine (GLT). ChE inhibitions are expressed as mean ± SD (*n* = 3 experiments).

Comp.	R	log *k*	IC_50_ (μM)		SI ^1^
			AChE	BChE	
**1**	H	0.1160	65.17 ± 2.24	175.18 ± 5.85	0.37
**2**	2-OCH_3_	0.3368	50.14 ± 1.57	22.23 ± 0.53	2.26
**3**	3-OCH_3_	0.1280	69.26 ± 2.00	361.80 ± 8.12	0.19
**4**	4-OCH_3_	0.1176	57.49 ± 0.08	153.98 ± 6.09	0.37
**5**	3-CH_3_	0.2725	70.23 ± 1.70	167.46 ± 10.21	0.42
**6**	2-F	0.3577	50.07 ± 0.06	70.84 ± 5.61	0.71
**7**	3-F	0.2303	66.60 ± 0.14	197.62 ± 1.36	0.34
**8**	4-F	0.3924	47.37 ± 0.61	105.82 ± 0.43	0.45
**9**	2-Cl	0.5188	41.86 ± 0.15	140.06 ± 0.35	0.30
**10**	3-Cl	0.6411	61.62 ± 1.74	203.54 ± 7.49	0.30
**11**	4-Cl	0.6399	78.20 ± 1.58	138.67 ± 6.86	0.56
**12**	2-CF_3_	0.4318	56.42 ± 1.26	33.59 ± 0.71	1.68
**13**	3-CF_3_	0.4477	70.67 ± 0.57	309.25 ± 8.06	0.23
**14**	4-CF_3_	0.7408	71.42 ± 5.04	136.95 ± 21.94	0.52
**15**	2-OCF_3_	0.6227	36.05 ± 1.48	44.31 ± 0.87	0.81
**16**	2,3-F	0.4705	62.42 ± 1.42	199.81 ± 7.00	0.31
**17**	2,4-F	0.1912	79.23 ± 1.27	180.31 ± 7.04	0.44
**18**	2,5-F	0.4012	58.31 ± 0.20	157.34 ± 3.11	0.37
**19**	2,6-F	-0.1205	64.31 ± 4.74	42.95 ± 2.13	1.50
**20**	3,5-F	0.4072	67.36 ± 5.24	208.34 ± 5.30	0.32
**21**	2,3-Cl	0.5497	88.78 ± 1.42	247.56 ± 14.95	0.36
**22**	2,5-Cl	0.6138	99.14 ± 0.20	141.20 ± 7.12	0.70
**23**	2,6-Cl	0.8125	76.74 ± 0.74	117.57 ± 1.11	0.65
**24**	3,4-Cl	0.6664	57.99 ± 5.83	185.06 ± 6.13	0.31
**25**	3,5-Cl	0.9084	50.62 ± 0.38	143.08 ± 3.69	0.35
**26**	2,4-Br	0.7366	94.89 ± 0.97	261.65 ± 5.10	0.36
**27**	3,5-CF_3_	0.9667	59.35 ± 3.38	145.68 ± 2.65	0.41
**28**	2,4,6-F	-0.0131	49.87 ± 0.09	31.03 ± 2.11	1.61
**29**	3,4,5-F	0.5169	62.27 ± 0.75	183.71 ± 0.75	0.34
**30**	2,4,5-Cl	0.9360	94.88 ± 1.78	174.98 ± 10.47	0.54
**31**	2,4,6-Cl	0.3679	93.09 ± 1.80	57.11 ± 1.72	1.63
**32**	2,4,6-Br	0.4427	65.92 ± 2.81	56.01 ± 0.58	1.18
CbzPAS	–	–	58.08 ± 1.04	136.85 ± 21.80	0.42
RIV	–	–	50.10 ± 3.08	19.95 ± 0.31	2.51
GLT	–	–	4.0 ± 0.13	7.96 ± 0.59	0.50

^1^ SI (index selectivity) = IC_50_ (AChE)/IC_50_ (BChE). Greater selectivity to BChE in bold.

## Data Availability

Not applicable.

## Supplementary Materials

The following are available online at https://www.mdpi.com/article/10.3390/ijms22073444/s1, Table S1: Theoretically estimated partition coefficient calculated by set of alternative methods for ring-substituted benzyl[4-(arylcarbamoyl)phenyl-3-hydroxy]carbamates **1**–**32**. Table S2: Predicted values—physicochemical properties of rivastigmine, galanthamine and compound **2**.
