# Supplementary material for: Synthesis and Hybrid SAR Property Modeling of Novel Cholinesterase Inhibitors
Source: Int J Mol Sci. 2021 Mar 26;22(7):3444. doi: 10.3390/ijms22073444 (PMC8037530; doi:10.3390/ijms22073444)
Supplement: Supplementary file 1 [file ijms-22-03444-s001.pdf]

Supplementary Materials

Article

Synthesis and Hybrid SAR Property Modeling of Novel Cholinesterase Inhibitors †

Jiri Kos [1,*], Violetta Kozik [2], Dominika Pindjakova [3], Timotej Jankech [3,4], Adam Smolinski [5], Sarka Stepankova [6,†], Jan Hosek [7], Michal Oravec [8], Josef Jampilek [1,3] and Andrzej Bak [2,*]

[1] Regional Centre of Advanced Technologies and Materials, Czech Advanced Technology and Research Institute, Palacky University, Slechtitelu 27, 78371 Olomouc, Czech Republic; josef.jampilek@gmail.com
[2] University of Silesia, Department of Chemistry, Szkolna 9, 40007 Katowice, Poland; violetta.kozik@us.edu.pl
[3] Department of Analytical Chemistry, Faculty of Natural Sciences, Comenius University, Ilkovicova 6, 842 15 Bratislava, Slovakia; pindjakova.dominika@gmail.com (D.P.); timotej.jankech@gmail.com (T.J.)
[4] NT-LAB o.z., Teplicka 35, 92101 Piestany, Slovakia
[5] GiG Research Institute, Pl. Gwarkow 1, 40166 Katowice, Poland; smolin@gig.katowice.pl
[6] Department of Biological and Biochemical Sciences, Faculty of Chemical Technology, University of Pardubice, Studentska 573, 53210 Pardubice, Czech Republic; sarka.stepankova@upce.cz
[7] Department of Pharmacology and Toxicology, Veterinary Research Institute, Hudcova 296/70, 62100 Brno, Czech Republic; hosek@vri.cz
[8] Global Change Research Institute CAS, Belidla 986/4a, 60300 Brno, Czech Republic; oravec.m@czechglobe.cz
* Correspondence: jiri.kos@upol.cz (J.K.); andrzej.bak@us.edu.pl (A.B.)

† This article is sincerely dedicated to the memory of Dr. Milan Hutta, a Professor of Analytical Chemistry at the Department of Analytical Chemistry, Faculty of Natural Sciences, Comenius University in Bratislava (Slovakia), a long-time head of the department, a versatile scientist and especially a rare person of outstanding character who left us these days due to the consequences of disease COVID-19 caused by the coronavirus SARS-CoV-2.

Table S1. Theoretically estimated partition coefficients calculated by set of alternative methods for ring-substituted benzyl [4-(arylcarbamoyl)phenyl-3-hydroxy]carbamates **1**–**32**.

No.	logP[a]	miLogP[b]	ClogP[c]	ClogP[d]	ClogP[e]	ClogP[f]	ClogP[g]	MlogP[h]	AlogP[i]	ClogP[j]	ClogP[k]	ClogP[l]	ClogP[m]
**1**	4.42	4.59	3.78	3.48	3.44	4.34	4.84	3.69	3.40	4.41	4.50	3.68	4.62
**2**	4.53	4.60	3.71	3.23	2.92	4.19	4.74	3.67	2.86	3.93	4.00	3.55	4.74
**3**	4.51	4.63	3.71	3.23	3.51	4.19	5.00	3.67	2.86	4.49	4.00	3.55	4.70
**4**	4.48	4.65	3.71	3.23	3.51	4.19	4.79	3.67	2.86	4.49	4.00	3.55	4.67
**5**	4.56	5.02	4.13	3.95	3.94	4.86	5.30	4.18	3.62	4.96	4.39	4.16	4.87
**6**	4.59	4.71	3.88	3.62	3.24	4.49	4.81	3.90	3.51	4.05	4.13	3.83	4.62
**7**	4.54	4.73	3.88	3.62	3.84	4.49	5.33	3.90	3.51	4.61	4.13	3.83	4.91
**8**	4.50	4.76	3.88	3.62	3.84	4.49	5.29	3.90	3.51	4.61	4.13	3.83	4.75
**9**	5.17	5.22	4.39	4.00	3.56	4.95	5.35	4.35	3.62	4.49	5.94	4.23	5.13
**10**	5.14	5.25	4.39	4.00	4.41	4.95	5.98	4.35	3.62	5.06	5.12	4.23	5.35
**11**	5.12	5.27	4.39	4.00	4.41	4.95	5.63	4.35	3.62	5.06	4.66	4.23	5.20
**12**	4.65	5.44	4.63	4.36	3.32	5.22	6.04	4.63	3.94	4.81	4.91	4.60	5.38
**13**	4.72	5.47	4.63	4.36	4.77	5.22	6.20	4.63	3.94	5.38	4.91	4.60	5.44
**14**	4.77	5.49	4.63	4.36	4.77	5.22	5.86	4.63	3.94	5.38	4.91	4.60	5.29
**15**	4.78	5.52	4.88	5.16	4.03	5.77	5.78	5.81	3.18	4.90	5.21	5.20	5.24
**16**	4.57	4.83	3.98	3.76	3.40	4.63	5.21	4.10	3.62	4.25	4.23	3.99	4.92
**17**	4.57	4.85	3.98	3.76	3.47	4.63	5.29	4.10	3.62	4.25	4.23	3.99	4.83
**18**	4.57	4.85	3.98	3.76	3.47	4.63	5.36	4.10	3.62	4.25	4.23	3.99	4.95
**19**	4.57	4.83	3.98	3.76	2.87	4.63	4.81	4.10	3.62	3.69	4.23	3.99	4.70
**20**	4.56	4.85	3.98	3.76	4.07	4.63	5.85	4.10	3.62	4.81	4.23	3.99	5.15
**21**	5.67	5.85	4.99	4.52	4.24	5.55	6.36	5.02	3.83	5.14	6.56	4.79	5.63
**22**	5.64	5.88	4.99	4.52	4.36	5.55	6.41	5.02	3.83	5.14	6.56	4.79	5.82
**23**	5.68	5.85	4.99	4.52	3.51	5.55	5.87	5.02	3.83	4.58	6.56	4.79	5.54
**24**	5.63	5.88	4.99	4.52	5.09	5.55	6.64	5.02	3.83	5.70	5.28	4.79	5.71
**25**	5.64	5.88	4.99	4.52	5.21	5.55	7.13	5.02	3.83	5.70	5.28	4.79	6.12
**26**	5.84	6.14	5.23	5.06	4.63	5.88	6.65	5.19	4.04	5.63	5.41	5.33	5.92
**27**	5.39	6.31	5.48	5.25	5.81	6.10	7.83	5.57	4.45	6.34	5.80	5.52	6.10
**28**	4.73	4.94	4.08	3.90	3.04	4.77	5.33	4.31	3.72	3.89	4.33	4.15	4.81
**29**	4.73	4.94	4.08	3.90	4.10	4.77	6.12	4.31	3.72	5.01	4.33	4.15	5.26
**30**	6.18	6.48	5.60	5.04	4.98	6.16	7.09	5.68	4.04	5.78	7.19	5.35	6.24
**31**	6.20	6.48	5.60	5.04	4.25	6.16	6.69	5.68	4.04	5.22	5.91	5.35	6.17
**32**	6.03	6.88	5.96	5.86	4.64	6.65	7.24	5.94	4.35	5.96	6.10	6.16	6.58

[a] clogPS, [b] Molinspirations, [c] OSIRIS property explorer, [d] HyperChem 7.0, [e] Sybyl-X, [f] Marvin Sketch (ChemAxon) 15, [g] ChemSketch 2015, [h] Dragon 6.0 , [i] Dragon 6.0, [j] Kowwin, [k] XlogP3, [l] ChemBio, [m] ACD/Percepta.

Table S2. Predicted values – physicochemical properties of rivastigmine, galanthamine and compound **2**. (Calculated using ACD/Percepta 2012 (Advanced Chemistry Development Inc., Toronto, ON, Canada)

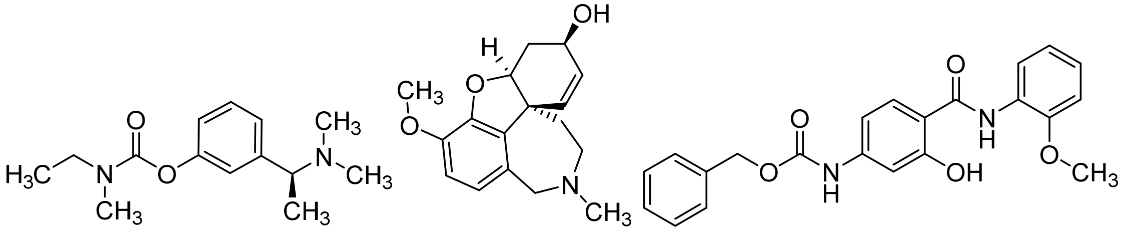

Parameters	rivastigmine	galanthamine	comp. 2
**MW**	250.34	287.35	392.40
**No. of H-bond donors**	0	1	3
**No. of H-bond acceptors**	4	4	7
**TPSA**	32.78	41.93	96.89
**No. of rotatable bonds**	5	1	7
**LogP**	2.29	1.55	4.74
**LogS$_w$**	-0.97	-1.83	-5.68
**C Ratio**	0.78	0.81	0.76
**N Ratio**	0.11	0.05	0.07
**NO Ratio**	0.22	0.19	0.24
**Hetero Ratio**	0.22	0.19	0.24
**Halogen Ratio**	0.00	0.00	0.00
**No.of Rings**	1	4	3
**No.of Aromatic Rings**	1	1	3
**No.of Rings (size 3)**	0	0	0
**No.of Rings (size 4)**	0	0	0
**No.of Rings (size 5)**	0	1	0
**No.of Rings (size 6)**	1	2	3
**BCF**	1.40	1.10	3.37
**KOC**	2.54	2.33	3.96
**DC**	0.00	0.00	0.00
**Parachor [cm^3]**	594.56	614.37	814.13
**Index of refraction**	1.52	1.64	1.68
**Surface tension [dyne/cm]**	36.95	56.64	61.36
**Density [g/cm^3]**	1.04	1.28	1.35
**Polarizability [cm^3]**	$28.99 \cdot 10^{-24}$	$31.84 \cdot 10^{-24}$	$43.57 \cdot 10^{-24}$
**Strongest pK$_a$ (acid)**	–	13.9 ± 0.2	7.9 ± 0.3
**Strongest pK$_a$ (base)**	8.6 ± 0.5	7.9 ± 0.4	-1.3 ± 0.7

Benzyl {3-hydroxy-4-[(2-methoxyphenyl)carbamoyl]phenyl}carbamate (**2**), one of the most effective compounds in the series and with the highest BChE selectivity of inhibition, has a lipophilicity expressed as logP approximately 1.5-fold higher than the clinically used drugs rivastigmine and galanthamine, which is a prerequisite for good absorption. On the other hand, compound **2** has a much lower logS$_w$ value and also the ability to protonate is completely different; while both drugs are basic in nature, carbamate **2** is rather acidic. Due to its structure, compound **2** has a much greater ability to form H-bonds. The polar surface area of carbamate **2** is almost 3-fold higher compared to rivastigmine and approximately twice as high as galanthamine. Compound **2** represents a flexible system closer to the properties of rivastigmine than the rigid structure of galanthamine. An almost 3-fold higher predicted bioaccumulation factor (BCF), defined as the ratio of compound concentration in tissue to concentration in the medium, means that compound **2** should have a higher tendency to be taken up by organs compared to both drugs. This fact corresponds to almost twice the adsorption coefficient (KOC) compared to two drugs discussed. Based on these software-predicted properties, it can be estimated that the prepared compound **2** meets the requirements for druglikeness and is able to be bound by molecular targets.
